# Exploring Trade-Offs between Fisheries and Conservation of the Vaquita Porpoise (*Phocoena sinu*s) Using an Atlantis Ecosystem Model

**DOI:** 10.1371/journal.pone.0042917

**Published:** 2012-08-15

**Authors:** Hem Nalini Morzaria-Luna, Cameron H. Ainsworth, Isaac C. Kaplan, Phillip S. Levin, Elizabeth A. Fulton

**Affiliations:** 1 Marine Resources Assessment Group Americas Incorporated, Seattle, Washington, United States of America; 2 Conservation Biology Division, Northwest Fisheries Science Center, National Marine Fisheries Service, National Oceanic and Atmospheric Administration, Seattle, Washington, United States of America; 3 Division of Marine and Atmospheric Research, The Commonwealth Scientific and Industrial Research Organisation Wealth from Oceans Flagship, Hobart, Tasmania, Australia; Aristotle University of Thessaloniki, Greece

## Abstract

**Background:**

Minimizing fishery bycatch threats might involve trade-offs between maintaining viable populations and economic benefits. Understanding these trade-offs can help managers reconcile conflicting goals. An example is a set of bycatch reduction measures for the Critically Endangered vaquita porpoise (*Phocoena sinus*), in the Northern Gulf of California, Mexico. The vaquita is an endemic species threatened with extinction by artisanal net bycatch within its limited range; in this area fisheries are the chief source of economic productivity.

**Methodology/Principal Findings:**

We analyze trade-offs between conservation of the vaquita and fisheries, using an end-to-end Atlantis ecosystem model for the Northern Gulf of California. Atlantis is a spatially-explicit model intended as a strategic tool to test alternative management strategies. We simulated increasingly restrictive fisheries regulations contained in the vaquita conservation plan: implementing progressively larger spatial management areas that exclude gillnets, shrimp driftnets and introduce a fishing gear that has no vaquita bycatch. We found that only the most extensive spatial management scenarios recovered the vaquita population above the threshold necessary to downlist the species from Critically Endangered. The scenario that excludes existing net gear from the 2008 area of vaquita distribution led to moderate decrease in net present value (US$ 42 million) relative to the best-performing scenario and a two-fold increase in the abundance of adult vaquita over the course of 30 years.

**Conclusions/Significance:**

Extended spatial management resulted in the highest recovery of the vaquita population. The economic cost of proposed management actions was unequally divided between fishing fleets; the loss of value from finfish gillnet fisheries was never recovered. Our analysis shows that managers will have to confront difficult trade-offs between management scenarios for vaquita conservation.

## Introduction

The incidental capture or entanglement of non-target species (“bycatch”) is a ubiquitous feature of marine fisheries, and is a major factor underlying the declines of marine megafuna worldwide [1]–[3]. In some situations eliminating all mortality associated with bycatch is an imperative to avert extinction [3]. This may take the form of modifying fishing gears or in some cases closing or limiting fisheries. However, many such restrictions on fisheries will result in at least short-term reductions in fishers' employment and incomes. Such economic impacts potentially lead to costly and socially divisive conflicts, particularly in regions that rely heavily on fishing and/or areas where alternative employment opportunities are poor [4]. Understanding the trade-offs between conservation benefits and economic costs is thus crucial for developing well-informed policies and management strategies [5].

Vaquita, *Phocoena sinus*, is a small porpoise endemic to the Upper Gulf of California, Mexico. The vaquita population is small (245 individuals in 2008; 95% CI 68–884; [6]), with a localized distribution that is coincident with large and highly profitable industrial and artisanal fisheries, mainly for blue shrimp (*Litopenaeus stylirostris*) [7]. Vaquita are entangled in gillnets that target finfish, and driftnets targeting shrimp; these gears account for the majority of bycatch mortality [8], [9]. Industrial shrimp trawling is also known to catch vaquita [10] and may disrupt vaquita behavior [8]. The combination of low fecundity and late maturity with high bycatch mortality [8] has resulted in vaquita being considered the world's most Critically Endangered cetacean [11].

Throughout the distribution of vaquita, the Mexican government is employing a set of economic incentives (Table 1) to eliminate shrimp driftnets and finfish gillnets [8], [9], [12] from the vaquita distribution area (see Text S1 for details), and has also reduced industrial trawl effort [13], [14]. These incentives would need to compensate for the current profitability of fishing activity; in the Upper Gulf catch in artisanal fisheries is valued at over US$ 10 million [7]. Economic analysis of the plan to eliminate nets from the vaquita distribution area estimated a 93% reduction in the total value of catch of shrimp driftnet and finfish gillnets fisheries, although it did not consider catch revenue from other gears or alternate economic activities [15].

**Table 1:** Economic incentives within the vaquita recovery program for small-scale fishers and fishing cooperatives.

Option		Requirements	Benefits
Rentout	Annual payment to stop fishing	Agree to stop fishing in the Vaquita refuge	∼$ 3, 500 USD‡
Switchout	Substitution of finfish gillnets and shrimp driftnets for gears with no vaquita bycatch	Turn in nets. Agree to use alternative gear.	∼$ 25, 000 USD†. New fishing permit.
Buyout	Fishermen stop fishing and are given a payment destined specifically for an alternate business or economic activity	Turn in boat, nets and permits	∼$ 25–35, 000 USD† (depending on number of permits)

Program is open to those based in the communities of San Felipe, in the state of Baja California, and in Golfo de Santa Clara and Puerto Peñasco, in the state of Sonora, Mexico, that own finfish gillnet and shrimp driftnet permits. Payments and guidelines for fiscal year 2011 [60].

‡ Yearly payment,

† One-time payment.

Ortiz [16] and Gerrodette and Rojas Bracho [17] analyzed the possibility of recovery of the vaquita using single-species models; they found that eliminating gillnets from the vaquita distribution area gave the most complete protection to vaquita, reducing the probability of extinction to 3% [16] and 2% respectively [17]. Likewise, applications of Ecopath with Ecosim ecosystem models [18], [19], which consider trophic interactions and dynamic modeling to explore the impacts of fishing [20], found negative impacts on vaquita from the high incidental mortality. However, there has been no concurrent analysis of the effects of management actions on vaquita and Upper Gulf fisheries [15].

An important advance in marine conservation has been the rise of ‘end-to-end” ecosystem models that integrate submodels of physical, biological and socio-economic components of the system [21]–[23]. These models have been particularly useful for exploring the ecological and conservation consequences of fisheries management scenarios, such as bycatch reduction programs. For instance, Hutton and colleagues [24] used an end-to-end model, built in the Atlantis modeling framework [21], [22], to explore the effects of bycatch quotas and bycatch penalties on marine mammals. Atlantis sets single species dynamics in the context of trophic interactions, multiple fleets, and spatial and temporal variability [22]. However, ecosystem models such as Atlantis are not meant to fully capture statistical uncertainty in all parameters, as would a single species model. For this reason, these models are meant for strategic evaluations (e.g., ranking performance of alternative policy options) and not as tactical management aids (e.g., for setting total allowable catch or gear restrictions) [21].

Here, we use an Atlantis ecosystem model of the Northern Gulf of California to evaluate alternate management actions outlined in the vaquita recovery plan [9] and proposed by the International Committee for the Recovery of the Vaquita in 2012 [25]. We evaluate effects on vaquita abundance and on the net economic benefit of fisheries catch. Changes in biodiversity and other ecological processes derived from these management actions are analyzed in a separate manuscript (Morzaria et al., *unpublished information*). Simulating the effect of management on vaquita can yield valuable insights, as experiments are costly and intensive monitoring is required to detect whether and at what rate the population is recovering [6], [26]. Our results illustrate potential trade-offs between fisheries and conservation. We found that only extensive spatial management scenarios recovered the vaquita population, at the cost of decreasing the net present value of fisheries.

## Methods

### Atlantis ecosystem model

We adapted the Atlantis model for the Northern Gulf of California, developed by Ainsworth et al. [27], [28]. Technical specifications of the Atlantis code base and a review of existing applications are detailed elsewhere (e.g. [22], [29]). In brief, Atlantis incorporates multiple submodels that simulate oceanography, biogeochemistry, food web interactions and human impacts, especially fisheries [30]. Species of particular ecological, management or conservation importance are represented with enough detail to evaluate direct effects of fishing, while other species are aggregated into functional groups with sufficient resolution to capture human, trophic, and climate impacts on the ecosystem [22].

The Northern Gulf of California Atlantis model was built to test ecosystem-based fisheries management questions and ecological hypotheses. The initial conditions of the model represent ecosystem structure and function for 2008 and provide a comprehensive representation of the Northern Gulf's oceanography, historical fishing patterns, migration and movement of key species, and variability in diet compositions, among other features. Ecosystem dynamics are simulated in 12-hour time steps. Model dynamics were calibrated against historical time series of abundance and catch from fisheries statistics, field data, and stock assessments available for the period from 1985–2008 (see Text S2 and [27] for details). Stock productivity and resilience were tested by a series of simulations ranging across fishing mortality rates. Unexploited biomasses of functional groups corresponded well to estimates by previous authors.

**Figure 1:**
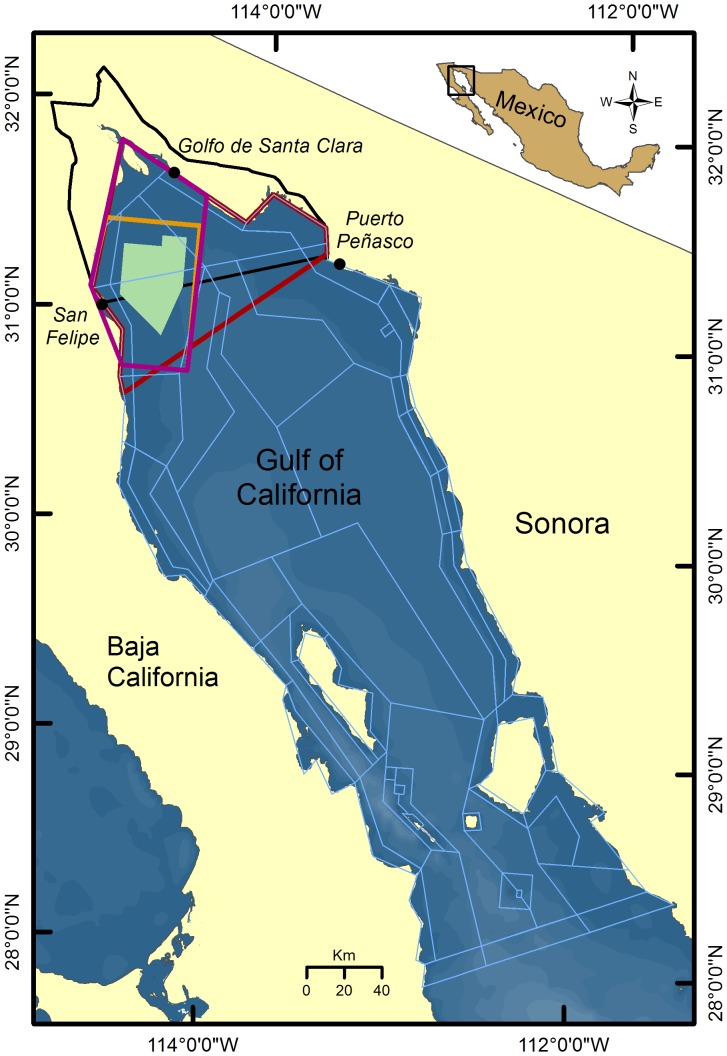
Northern Gulf of California and Atlantis model extent. Atlantis polygon geometry (blue lines), Biosphere Reserve (dark black line), vaquita refuge and core (green), extended refuge (orange) and fishing communities in the vaquita distribution area (red).

The model extends over 57800 km^2^, from the Colorado River Delta south to the northern tip of Baja California Sur (Fig. 1). The model area is divided into 66 polygons based on ecologically important gradients and boundaries, stock assessments, catch data, and spatial management boundaries. Individual polygons include one sediment layer and up to six water depth layers. Biological, chemical and physical processes replicated within each spatial cell drive the model. Fluxes of water, heat and salt are forced by a Regional Oceanographic Model System (ROMS) [31]. Water flux drives the advection of plankton, nutrients and waste cycling; temperature affects growth, consumption and primary production rates. The spatial distribution of abundance for the 63 functional groups is defined per model polygon and depth layer.

Functional group dynamics are driven by formulations that describe biological processes, including consumption, reproduction, waste cycling, predation, recruitment, habitat dependency, mortality, growth, and movement (both foraging behavior and horizontal/vertical migration). Some key assumptions include density dependent movement, where predators move according to abundance of prey items; reproduction based on Beverton-Holt stock recruitment relationships (for fish except elasmobranchs) and fixed reproduction per female for elasmobranchs, mammals, birds, and turtles; predation calculated by a Holling Type II functional response, where diet compositions vary through time and feeding rates vary dynamically according to gape limitation; and dynamic weight at-age, where dynamic consumption rates result in variable weight at-age. For vertebrates, we track abundance and size-at-age per age class. For invertebrates, we track biomass pools. Unlike in single-species models, in Atlantis birth and death rates are not fixed, but rather evolve as predation, growth and fecundity change through time.

Vaquita age structure is divided in ten classes, with a maximum longevity of 20 years; they are considered sexually mature at age ten [32]. Average body weight was 22.3 kg vaquita^−1^ [33]. Diet composition for vaquita was based on averaged values from previous ecosystem models for the Northern Gulf [19], [34], and is dominated by small pelagic fish (Table S1), although modeled diets will vary through time and space based on prey availability. Diet for juvenile vaquita was assumed similar to adults. Large pelagic sharks are considered the only predators on vaquita [10]. The model uses a fixed recruitment relationship for vaquita, in which each female is assumed to birth 0.26 viable offspring per year [27]. The initial distribution of vaquita spatial abundance follows Gerrodette et al. [6], [35] (Fig. S1). Vaquita newborns and all older age classes are subject to fishing mortality by all fleets in the model [36], insufficient data was available to specify age selectivity by gears (but see [36], [37]). We set initial vaquita mortality at 0.15 year^−1^ under No management (see below *Scenarios*) the median estimate in 2007 [17], before the implementation of bycatch reduction measures. When vaquita dynamics in Atlantis are tested against the historical vaquita abundance trajectory for the period 1993–2008, model results are within the 95% confidence intervals for published abundance estimates [6], [38], [39] (see Text S2 for details).

### Scenarios

We projected five management scenarios over 30 years, from 2008 to 2038. The scenarios described here began with the same initial parameterization of ecology and oceanography; thus, the differences in outcomes result from the direct and indirect effects of fishing. Fishing mortality is imposed by the fishing fleets onto all relevant target and bycatch functional groups.

We first simulated a reference scenario (No management scenario) that did not include any management actions for vaquita protection. This scenario includes the current degree of compliance with existing fisheries restrictions (see Ainsworth et al. [28] for details); it incorporates a 30% reduction in trawl effort within the Upper Gulf Biosphere Reserve (Fig. 1) implemented in 2008 [13]. We based initial (2008) catches of species other than vaquita on the average of 2000–2007 catches, assembled from official fishery statistics, port-level surveys, and fisher log books (Table S2). We used data on fleet and gear catch composition to assign a proportion of the catch to thirty-three fleets in the model (Tables S3 and S4); each of these fleets has specific fishing areas (see Ainsworth et al. [28] for details). We set vaquita mortality at 0.15 year^−1^, the median estimate for 2007 [17], before the implementation of bycatch reduction measures. Although industrial shrimp trawlers have been reported to catch vaquitas, there are no fleet estimates of bycatch mortality [10], therefore we allocated all vaquita bycatch to shrimp driftnet and finfish gillnet fleets. Allocation of vaquita bycatch between gillnet fleets was based on the number of fishing permits in 2008 per fleet [40] as a measure of effort, as there appears to be no difference in bycatch mortality between different fisheries and mesh sizes [37].

The No management scenario was then compared to four scenarios that simulate management actions in the species recovery plan [9], mitigation measures in the Environmental Impact Assessment for industrial shrimp trawling within the Upper Gulf Biosphere Reserve [13], [14] and the latest recommendations by the International Committee for the Recovery of the Vaquita [25]. The scenarios combine spatial closures for industrial shrimp trawls and gillnets while allowing the shrimp driftnet fleet to switch to a light trawl instead of being excluded. The spatial closures occur in the area where vaquita sightings are concentrated [38]. We began all scenarios using the most recent estimate of vaquita abundance (245; [6]), and complemented these with scenarios initialized at the upper and lower limits of the 95% confidence interval (68–884). We used this approach to estimate uncertainty derived from initial population sizes since Atlantis is a deterministic model and computational limits prevent extensive stochastic realizations.

The four scenarios (Fig. 1) include a 1264-km^2^ spatial closure to industrial shrimp trawls within the current refuge [13], [14]. The scenarios also include progressively larger spatial closures for shrimp driftnets and finfish gillnets: 1) The Vaquita refuge scenario includes a 1264-km^2^ spatial closure [9], [13], representing the 2010 status quo (see Supporting Information for details). 2) The Extended refuge scenario is a 3579-km^2^ spatial closure representing an option from the species recovery plan [9]. 3) The Primary area scenario excludes nets from a refuge encompassing the vaquita distribution as of 2008 (5339 km^2^) [6], corresponding to the recommendation of the International Committee for the Recovery of the Vaquita in 2012 [25]. 4) The Distribution area scenario closes off the entire known vaquita range (8432 km^2^) and is equivalent to the 2012 target in the species recovery plan [9]. The closures were simulated as partial or complete spatial closures to model cells affected for the corresponding fleets, with fishing effort reduced proportionally to area closed. The reconversion program being implemented to eliminate vaquita bycatch is designed to minimize redistribution of fishing effort (Table 1); we did not consider possible increases in illegal fishing.

In each of these four scenarios a new light shrimp trawl fleet that eliminates vaquita bycatch [41] was allowed to operate within the spatial closure area. We assumed that as the area closed to shrimp driftnets and finfish gillnets increases, adoption of the light trawl will increase, as many fishers want to continue fishing [7], shrimp are profitable [42], and removal of driftnets and gillnets facilitates the use of light trawl by reducing chances of entanglement. This is consistent with a recent analysis which found that the fishers which opted for the buyout were those close to retirement and that no fishers have opted to leave the fishery since 2010 [12]. Our analysis does not consider participation in the rentout option (Table 1). Shrimp catch rates for the light trawl may be dependent on the level of training and skill by fishers [25], [43], varying from reductions of 13% [43] to 56% [44] relative to the shrimp driftnet. Therefore, we ran each spatial closure scenario considering 10%, 20%, 30%, 40% and 50% reductions in shrimp catch, to assess the variation in net profits. Based on results from recent trials we set the shrimp:bycatch ratio of 1.37 for the light trawl fleet [43] compared to a ratio of 1.23 for the shrimp driftnet [45] (bycatch is primarily fish, and not vaquita). Bycatch composition of the light trawl fleet also differed from that of the shrimp driftnet fleet ([41], [45]; Table S5). Fishing mortality of vaquita was set to 0 for this fleet.

### Assessment of economic costs

To assess the economic cost of management actions, we calculated net economic benefit (NB) per functional group as,
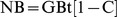
where GB is gross benefit (i.e. value of catch) for year *t* and *C* is cost rate for fishing. We assumed a cost rate of fishing of 32% for artisanal fleets [7] and a base cost of 96% for industrial fleets (Table S6). Prices were determined from the average 2005–2010 value, dollars tonne^−1^, by functional group (Table S7).

We then calculated the net present value (NPV) of profits to commercial fisheries by discounting the net benefit over time with a standardized rate [46]. Discounting implies that $1 received at some time in the future is perceived to be worth less to a person than $1 received now, and reflects uncertainty and lost opportunity costs. By calculating NPV, a common metric in cost-benefit analysis, the economic benefits of all scenarios are standardized and are comparable against other alternative investments that a fisher could receive, such as earning bank interest. NPV was calculated as
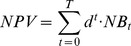
where NB is the sum of net benefits accruing in year *t* for all functional groups and *d* is the discount factor,
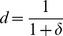
As a discount rate (δ), we used the 2000–2010 Mexican government bond real interest rate of 3.6% (Center for the Study of Public Finance, House of Representatives).

For industrial shrimp trawl fleets, we only included shrimp and blue crab catch in calculation of value and assumed all other groups were discarded [47]. We present net benefit separately for industrial and artisanal fleets, and by gear (grouped fleets), to make it easier to explain the effects of the simulated management actions. Results for the end of the 30-year simulations are presented as the average of the last five years, 2034–2038 (± SE) to emphasize differences amongst scenarios rather than temporal trends which are dominated by underlying biomass dynamics.

## Results

### Vaquita recovery

**Figure 2:**
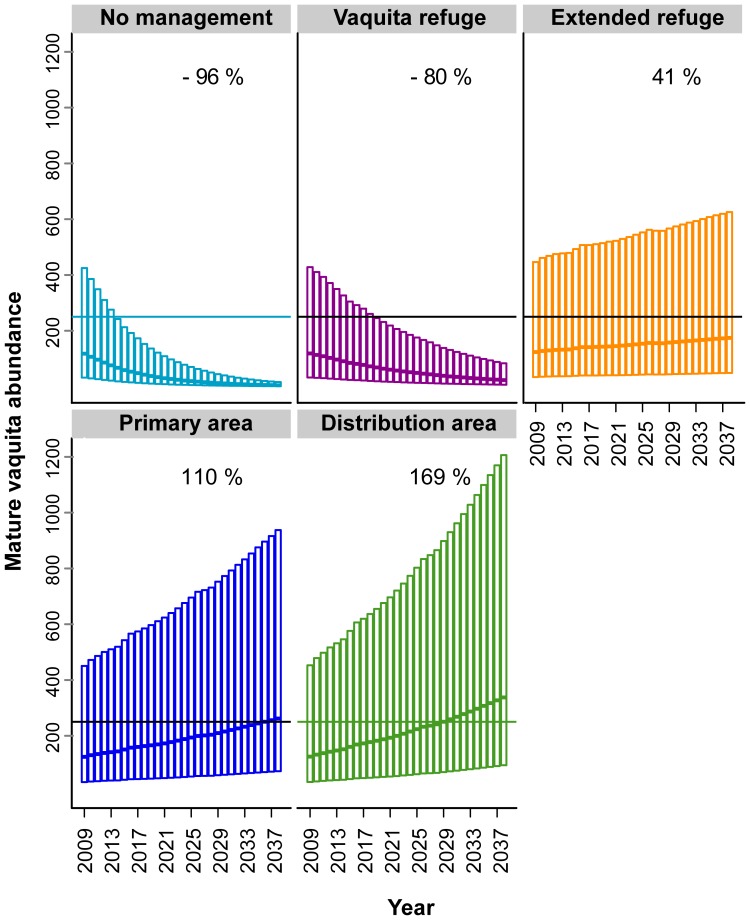
Mature vaquita abundance trajectories from 2009 to 2038 for each management scenario. Dark lines are abundance assuming an initial vaquita population of 245 individuals [6]. Bars are simulation results for 95% CI interval estimates for vaquita abundance, top of bars is an initial 884 vaquitas and bottom of bars is an initial 68 vaquitas. Numbers are percent change in abundance relative to 2009, considering an initial abundance of 245 vaquitas. Solid line indicates 250 mature individuals, a criterion for downlisting from the Critically Endangered category of the IUCN Red List of Threatened Species™ [11].

Mature vaquita abundance trajectories for each scenario, across the 30-year simulations, are shown in Fig. 2. In the No management scenario and in the Vaquita refuge scenario that included a 1264 km^2^ spatial closure, abundance showed a downward trend regardless of the initial vaquita abundance. For these scenarios, mature vaquita abundance in year 30 decreased significantly (−96%, No management; −80%, Vaquita refuge) relative to the start of the simulations. Given the strategic nature of Atlantis and the lack of demographic stochasticity in the model, the species can be considered functionally extinct in simulations where the population decreased and remained at low numbers for long periods. Atlantis does not include a population viability threshold but single-species models have found that persistent vaquita bycatch mortality would drive the population extinct [16], [17].

Only scenarios with large spatial closures led to a sustained increase in vaquita. The highest increase occurred in the Distribution area scenario, where mature vaquita abundance in year 30 was nearly 3 times higher than 2008, considering an initial abundance of 245 vaquitas. Even in these scenarios that reduce vaquita bycatch, mature vaquita abundance remained under the population threshold necessary to downlist the species from critically endangered [11] for the first 20 years of the simulations except when considering an initial abundance of 884 individuals (upper 95% CI on 2008 abundance; [17]); this pattern emphasizes that current rates of bycatch mortality are unsustainable [8].

**Figure 3:**
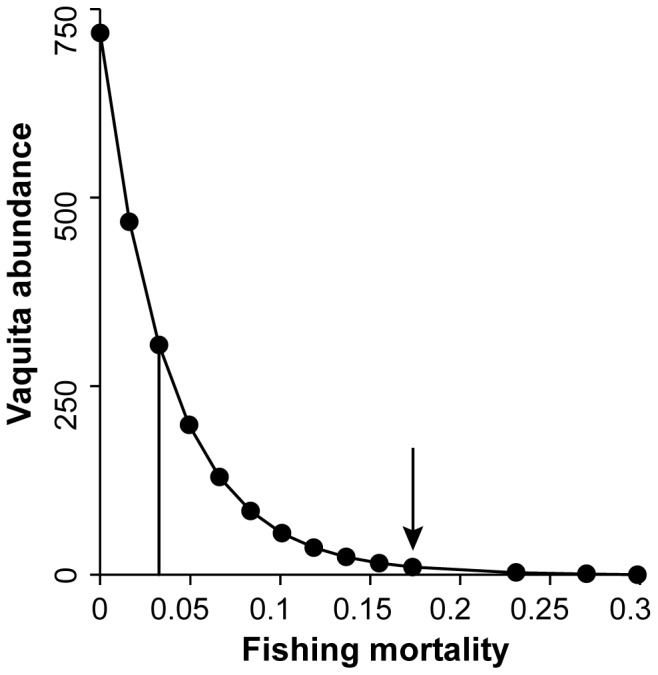
Vaquita abundance equilibrium curve. Vaquita abundance as a function of absolute bycatch mortality. Each bycatch mortality point in the figure is the ratio of average catch and biomass for the last 5 years (2033–2038) of a simulation run under a specific bycatch mortality value. Solid vertical line indicates maximum sustainable bycatch mortality. Arrow indicates bycatch mortality for the No management scenario.

We constructed a vaquita abundance equilibrium curve (Fig. 3) to further analyze population dynamics under varying rates of bycatch mortality. This equilibrium curve assumed deterministic population behavior in growth, recruitment and mortality; however it also accounted for species interactions, which can affect population dynamics in unexpected ways [48]. As expected, abundance of vaquita was highest under zero fishing effort. The model predicted an ecological carrying capacity of 773 individuals. This predicted carrying capacity is on the low range of a few thousand individuals suggested by the genetic analysis [49]. We found that the population could withstand bycatch mortality of 0.03 year^−1^, without vaquita abundance decreasing relative to initial abundance over the 30-year simulation. Bycatch mortality under the No management scenario, 0.17 year ^−1^, was more than five times this level of bycatch mortality that sustains the population at the current level.

**Figure 4:**
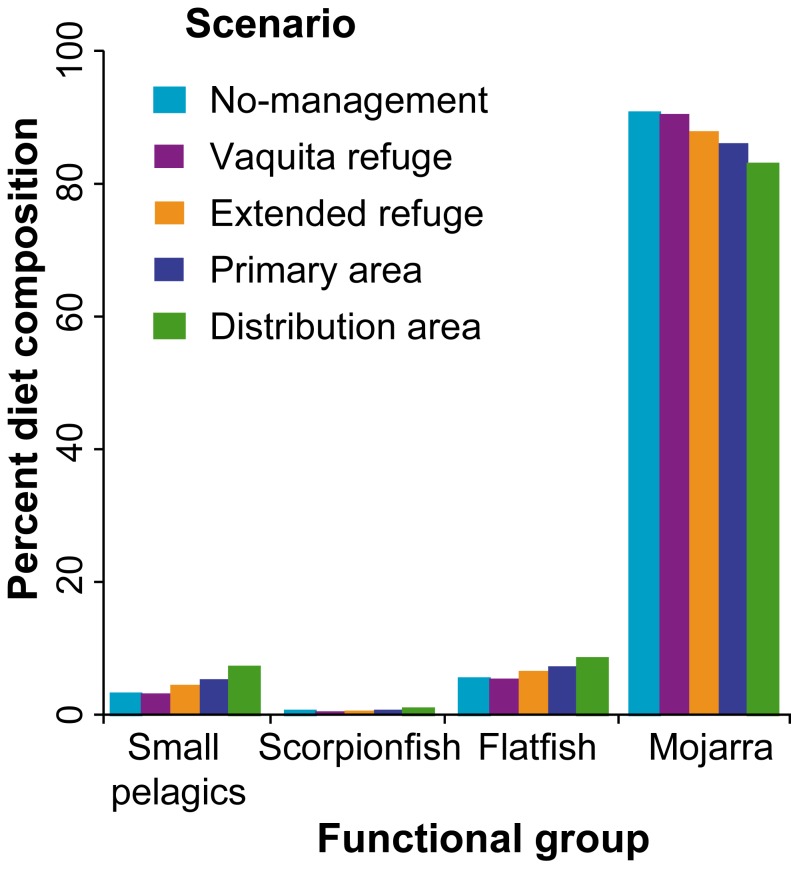
Vaquita prey composition for each management scenario. Prey that compose 99% of vaquita diet are shown.

In addition to changes in abundance, vaquita prey composition changed across management scenarios. Although vaquita still consumed the same 15 prey groups (Table S1), by year 30 diet proportions had changed (Fig. 4). In the most restrictive scenario, Distribution area, there was a decrease in mojarra (−8%) consumption relative to No management and increased predation by vaquita on small pelagic fish (132%), scorpionfish (71%), and flatfish (57%). Abundance of the groups that comprise less than 1% of vaquita diet also increased significantly in the Distribution area scenario relative to No management, including crabs and lobsters (323%), squid (142%), and totoaba (102%). Biomass of these prey groups increases as a result of decreased fishing pressure within spatial management areas.

### Fishery value

We first show results for net benefit, considering a 10% reduction in catch of the shrimp light trawl fleet relative to the driftnet fleet. We also analyze the effect of the range of reductions (10–50%) when examining NPV.

**Figure 5:**
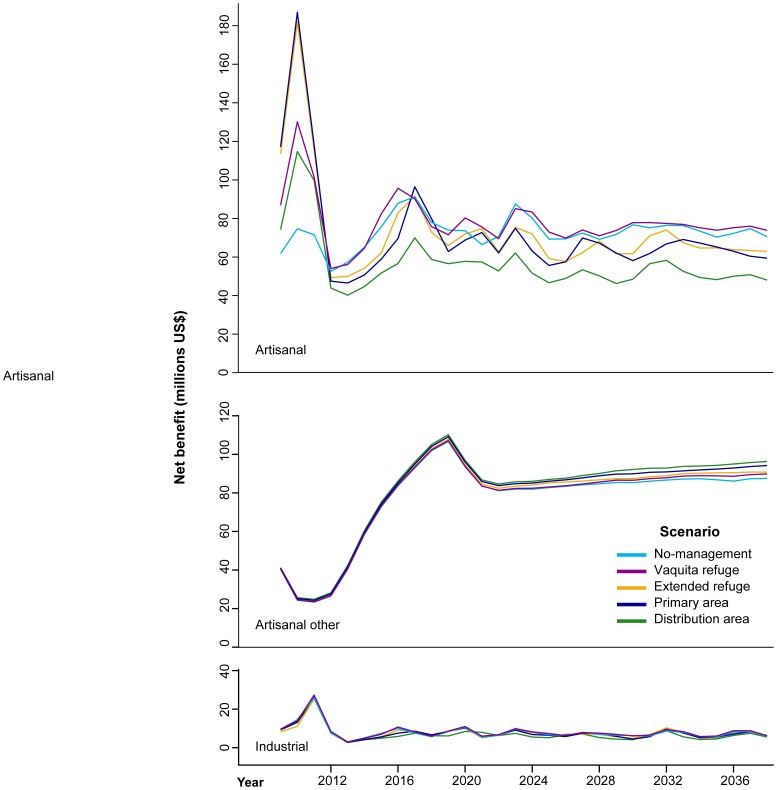
Undiscounted net benefit trajectories from 2009 to 2038 for each management scenario, considering a 10% reduction in shrimp catch for the light trawl fleet relative to the shrimp driftnet.

Temporal trends in the undiscounted annual net benefit of fisheries were dominated by underlying biomass dynamics (Fig. 5). For artisanal net fleets (finfish gillnets, shrimp driftnets and shrimp light trawl), which are directly affected by simulated management actions, net benefit increased the most in the No management scenario over the 30-year simulation (17% relative to 2009), to an average of US$72±0.8 million annually over the last five years of the simulation. However, the Vaquita refuge was the highest-value scenario by the end of the simulation, 4% higher than No management (equivalent to US$3 million). In this scenario, both the finfish gillnet and shrimp driftnet fleets still operate over a large area while the shrimp light trawl is being introduced. Nonetheless, as the area of spatial restrictions over gillnets and driftnet increases, net benefit decreases. In the most restrictive scenario, at the end of the simulation, average annual net benefit is US$23 million lower than No-management (Distribution area; US$49±0.5 million). Fishery gains from spatial management are modest because model-wide net benefit is driven by abundant finfish (over 65% of net benefit across scenarios) rather than harvest of sedentary species and overfished species more likely to benefit from reserves (Fig. S2). However, the contribution of different functional groups to net benefit changes with spatial management; target groups that decrease relative to No-management include Gulf grouper, large pelagics, and drums and croakers (Fig. S3). A greater proportion of catch value under spatial management comes from herbivorous fish and sharks.

Other artisanal fleets, including longline, handline, traps, and diving, benefit from spatial management. For all scenarios, average net benefit to these other artisanal fleets at the end of the simulations outperformed No management by US$ 2–8 million (Fig. 5). The highest-value scenario by the end of the simulation was the Distribution area (US$95±0.4 million). These gains in net benefit are a result of higher catch from groups that experience decreased fishing pressure from gillnet fleets through spatial restrictions, including herbivorous fish, sharks, Amarillo snapper and drums and croakers (Fig. S3).

There was a modest decrease in the net benefit of industrial fleets with spatial management, <US$1 million annually in every case. On average, Net benefit of industrial fisheries in the last five years of the simulation was highest under the Vaquita refuge scenario (US$7.1±0.7 million); but this was only 5% higher than No management. The losses that industrial fleets experience under spatial management are due to trophic effects. Net benefit (profits) from harvesting main target groups including shrimp (−23%) and small pelagics (−53%) decrease under spatial management relative to No management (Fig. S3), as a result of increased predation pressure (Fig. S4).

**Figure 6:**
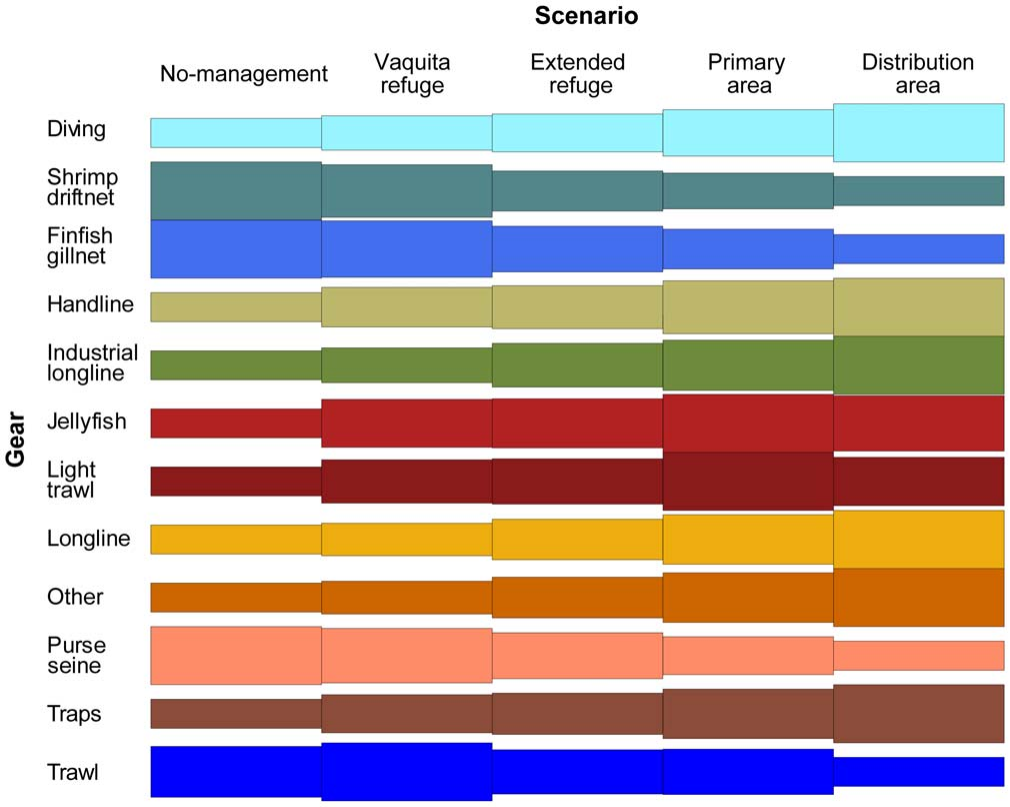
Comparison of undiscounted net benefit for gears between management scenarios. The tile plots shows average net benefit (value of catch minus costs) for gears (aggregated fleets) for the last five years of the 30 year simulation for each management scenario. The net benefit for each gear is scaled between the scenario with the highest and lowest value of catch.

We assessed how the distribution of average undiscounted net benefits among gears (grouped fleets) for the last five years of the simulations compared amongst scenarios (Fig. 6). Net benefit for all gears except the shrimp driftnet, gillnets, and purse seine, was lower in No management than in one or more other scenarios that allowed additional stocks to rebuild and led to subsequent increases in catch. Several gears including diving, handline, traps, and longline had highest value of catch under the restrictive Distribution area scenario.

**Figure 7:**
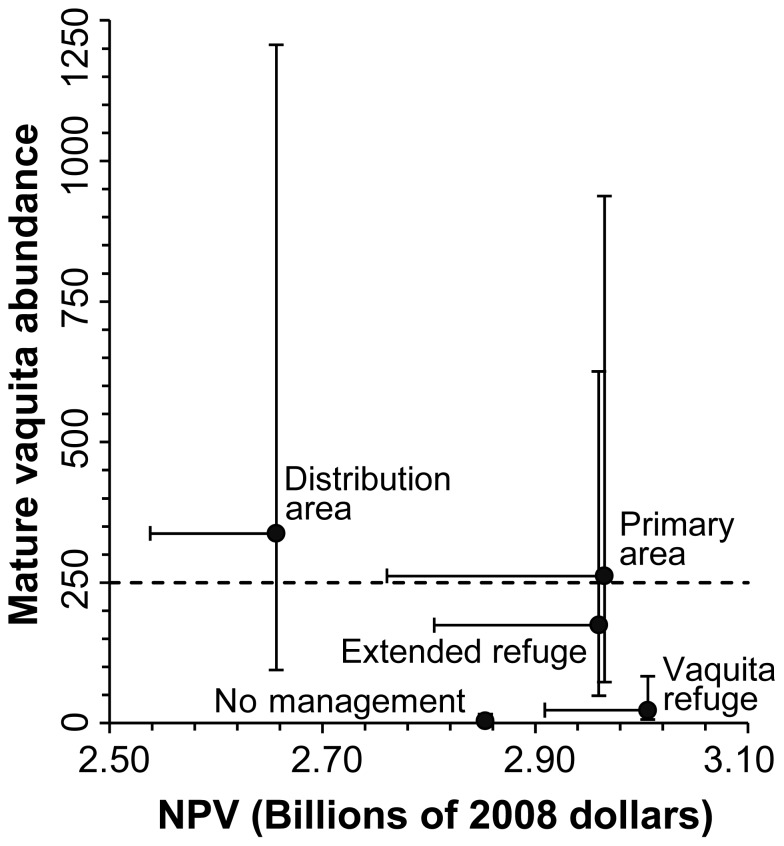
Net present value of fisheries catch for each management scenario, plotted against mature vaquita abundance at the end of the 30 year simulation. Net present value is discounted benefit over time with an alternate rate of return from bank investment (δ = 3.6%). Markers are results for initial vaquita abundance of 245 individuals and a 10% reduction in shrimp catch in the light trawl relative to the shrimp driftnet. Vertical confidence intervals are mature vaquita abundance results for initial abundance of 68 and 884 and horizontal intervals show 50% reductions in shrimp catch. Dashed line indicates 250 mature individuals, a criterion for downlisting from the Critically Endangered category of the IUCN Red List of Threatened Species™ [11].

Summing over all fleets, there was an inverse relationship between NPV and the abundance of mature vaquita. In scenarios without extensive spatial management (No management and Vaquita refuge), mature vaquita abundance remained well below the population threshold necessary to downlist the species from Critically Endangered [11]. NPV was lower in No management than all scenarios with spatial management except Distribution area (Fig. 7). The maximum NPV from fisheries, US$ 3billion, was achieved under the Vaquita refuge scenario. This value represents the discounted profit stream over the next 30 years. As the area under spatial management increased relative to the Vaquita refuge, NPV decreased 1–12% primarily due to declines in catch from net fleets. The distribution of NPV values suggests there may be convex relationship between NPV and mature vaquita abundance, possibly indicating a Pareto efficiency where joint benefits are maximized. The Primary area scenario, which represents the current recommendation of the International Committee for the Recovery of the Vaquita [25], could be close to this maximum. In this scenario NPV was US$ 2.96 billion and mature vaquita abundance was above the population threshold considering an initial abundance of 245 vaquitas.

We found that fishers' experience and training in operating the shrimp light trawl could affect NPV [25], [43].The 50% reduction in shrimp catch of the light trawl fleet, relative to the shrimp driftnet fleet, could account for US$193 million (Primary area) to US$ 98 million (Vaquita refuge) in lost NPV, relative to the best-case scenario (10% reduction; Table 2).

**Table 2:** Net present value (NPV; billions of 2008 dollars per year) for spatial management scenarios under various reductions of shrimp catch.

Scenario	Reduction in shrimp catch				
	10%	20%	30%	40%	50%
Vaquita refuge	3.006	2.982	2.958	2.934	2.909
Extended refuge	2.960	2.922	2.882	2.844	2.805
Primary area	2.965	2.913	2.867	2.820	2.761
Distribution area	2.657	2.628	2.599	2.570	2.538

We applied a range of reductions in shrimp catch in the light trawl fleet relative to the shrimp driftnet fleet (10%–50%), as shrimp catch in the light trawl will be dependent on fishers' experience and training [25], [43]. NPV for the No management scenario was 2.853.

## Discussion

Our study illustrates potential trade-offs between economic and conservation objectives when reducing bycatch threats for an endangered cetacean. Trade-offs are inherent in conservation, as any actions intended to reduce pressures from development or resource extraction will involve costs to local inhabitants that rely on those activities [50]; nonetheless trade-offs are rarely recognized or debated explicitly [51]. The rhetoric of win-win solutions underlies many popular conservation programs, although in practice gains for both biodiversity and human well-being are difficult to attain [52]. For example, the harvest of non-timber forest products was long thought to be both more profitable than other forest uses such as logging or agriculture and less ecologically destructive, but recent analyses show that there is uncertainty on the impact of non-timber forest product harvesting and on income generation [50].

In marine environments, there is empirical [53] and theoretical evidence [54] that spatial closures can result in win-win solutions, with both gains for conservation and increased economic benefits from fishery enhancement, as fish spillover from reserves to harvested areas. We found a more complex result. Our model results suggest that the Primary area scenario, which excludes finfish gillnets and shrimp driftnets from the vaquita distribution area as of 2008 (5339 km^2^) [6] could be a good compromise between vaquita conservation and fisheries. Under this scenario mature vaquita increase two-fold relative to 2008 while bycatch mortality remained at 0.03 year^−1^, equal to the maximum that the population could withstand without decreasing abundance over the 30 year simulation. From a fisheries perspective, NPV in the Primary area scenario decreased moderately (US$ 42 million) relative to the best-performing scenario. Nonetheless, the outcomes for individual fisheries varied. Finfish gillnet fisheries experienced large losses in net benefit under spatial management, while longline, handline, traps, and dive fisheries benefited from spatial management and net benefit of industrial fisheries decreased slightly. Therefore, the economic cost of proposed management actions will be unequally partitioned within the fishing community. Fishers will likely respond in changing fishing tactics and strategies, within (or outside) the regulatory framework, according to the type of alternate fishery available, fisher experience, risk perception and opportunity cost [55].

Our results agree with previous authors [16], [17], [19], [56] who concluded that preventing extinction of vaquita would require eliminating all nets and trawls from its distribution area or known range; this promises to be an expensive, politically unpopular process that has not been tried elsewhere on this scale [3], and would likely have high social and cultural costs [7]. Vaquita recovery will therefore be contingent on implementation of management actions, particularly economic alternatives provided to fishers [7], [57]. In a policy context, the value of those alternative income streams can be measured against the expected benefits (NPVs) provided here.

As with vaquita, many conservation projects around the world center on endangered and threatened species [1]; where preventing extinction of one species is costly and the outcome is uncertain. Nonetheless there are examples, such as the Steller sea lion in the North Pacific [58] where the risk of extinction for the population has declined following the implementation of management measures that restricted human activities. Incorporating an evaluation of associated ecosystem-level benefits, the probability of success, and the opportunity costs of conservation actions (i.e. alternative goals that could be achieved with the same resources) would help managers reconcile potentially conflicting benefits and values [59]. Ultimately, as in this case, society may often be required to confront the hard choices between protection of charismatic species and local livelihoods; end-to-end models such as the one applied here will be useful to evaluate the trade-offs.

## Supporting Information

Figure S1 **Initial vaquita spatial abundance distribution considering 245 individuals.** Grey lines are Atlantis polygon geometry. Abundance per polygon changes dynamically during simulations dependent on predator-prey and local habitat influences.
(TIF)

Figure S2 **Undiscounted net benefit for each gear and management scenario.** Values are averages for the last 5 years of the 30-year simulations (± SE). (*) indicates the scenario with the highest value for each gear. See text for calculation of net benefit.
(TIF)

Figure S3 **Percent change in undiscounted net benefit deriving from harvest of selected functional groups across scenarios, relative to No management.** Net benefit is average for the last 5 years of the 30-year simulations.
(TIF)

Figure S4 **Percent increase in predation for selected target groups across scenarios relative to No management, in the last year of the 30-year simulation.**
(TIF)

Text S1 **Management actions for vaquita conservation.**
(DOCX)

Text S2 **Historical vaquita abundance trajectory (1993–2008).**
(DOCX)

Table S1 **Vaquita diet based on averaged values from the ecosystem models developed by Morales-Zárate** [**1**] **and Lozano** [**2**] **; based on data analyzed by** [**3**], [**4**] **.** Diet for juveniles was assumed similar to adults. Values are proportion of total diet for each functional group used at the start of the simulations, realized diets will vary through time and space based on prey availability.
(DOCX)

Table S2 **Catch by functional group used as baseline in the No management scenario.** Catches are the average of the 2000–2007 model catch series from Ainsworth et al. [1] summed for all fleets and modified as described in Ainsworth et al. [2]. Vaquita mortality was set at 0.15 year-1, the median estimate prior to 2007 [3]. Since the publication of Ainsworth et al. [1], the model has been simplified to only include catch for a generic Penaeid shrimp group rather than for separate shrimp groups. Ainsworth et al. [1] provide species composition for each functional group.
(DOCX)

Table S3 **Fishery fleets in the Atlantis Northern Gulf of California model.** Fleets are defined based on gears, targets, bycatch, home ports and fishery utilization areas. Table indicates ports, functional groups targeted out of 63 in the model and gears. See Ainsworth et al. [1] and Ainsworth [2] for more details on fleets and functional groups used.
(DOCX)

Table S4 **Catch per fleet used as a baseline. Asterisk indicates group is bycatch; fleet name is in bold.** Vaquita catch was updated considering a mortality rate of 0.15 year-1. Bycatch composition for the Upper Gulf shrimp driftnet fleet was updated using more monitoring data [1]. Otherwise catch per fleet values are unmodified from Ainsworth et al. [2], [3].
(DOCX)

Table S5 **Catch for the shrimp light trawl fleet.** Bycatch composition was based on INAPESCA & NMFS [1] and considering a reduction in shrimp catch of 10% [1] and an increase in the ratio of shrimp to bycatch (other than vaquita) of 11% [1], [2]. Vaquita bycatch was set to 0 for this fleet.
(DOCX)

Table S6 **Sources for estimation of industrial fleets' cost rate of in the Northern Gulf of California.** Average cost rate of 0.956 was used to calculate net present value.
(DOCX)

Table S7 **Price matrix for Atlantis functional groups.** Values are dollars tonne-1 for 2010 or the most recent year for which data was available. For Penaeid shrimp, prices were set by fleet, weighted by the amount of blue, brown and Japanese shrimp caught. Information from National statistics for Sonora and Baja California, (Anuarios Estadísticos www.inegi.org.mx), state statistics for Sonora (www.oeidrus-sonora.gob.mx/), and port-level data for both states (unpublished data, A. Cinti, The University of Arizona, acinti@email.arizona.edu). Values were converted from Mexican pesos to dollars using the exchange rate from 2005–2010 (www.x-rates.com).
(DOCX)
