# Supplementary figures and images for: Exploring Trade-Offs between Fisheries and Conservation of the Vaquita Porpoise (Phocoena sinus) Using an Atlantis Ecosystem Model
Source: PLoS One. 2012 Aug 15;7(8):e42917. doi: 10.1371/journal.pone.0042917 (PMC3419746; doi:10.1371/journal.pone.0042917)

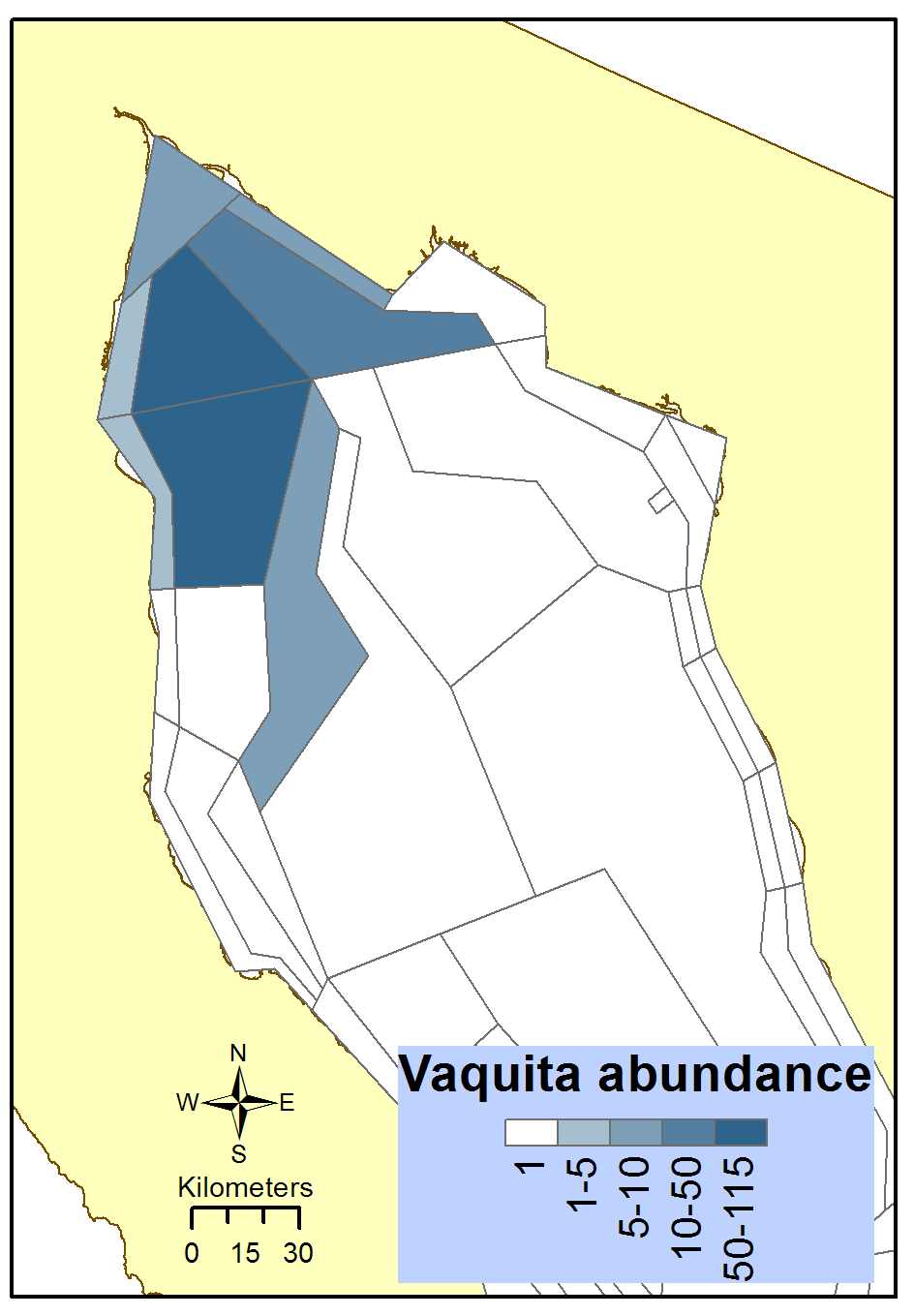

Supplement: Figure S1 — Initial vaquita spatial abundance distribution considering 245 individuals. Grey lines are Atlantis polygon geometry. Abundance per polygon changes dynamically during simulations dependent on predator-prey and local habitat influences. (TIF) [file pone.0042917.s001.tif]

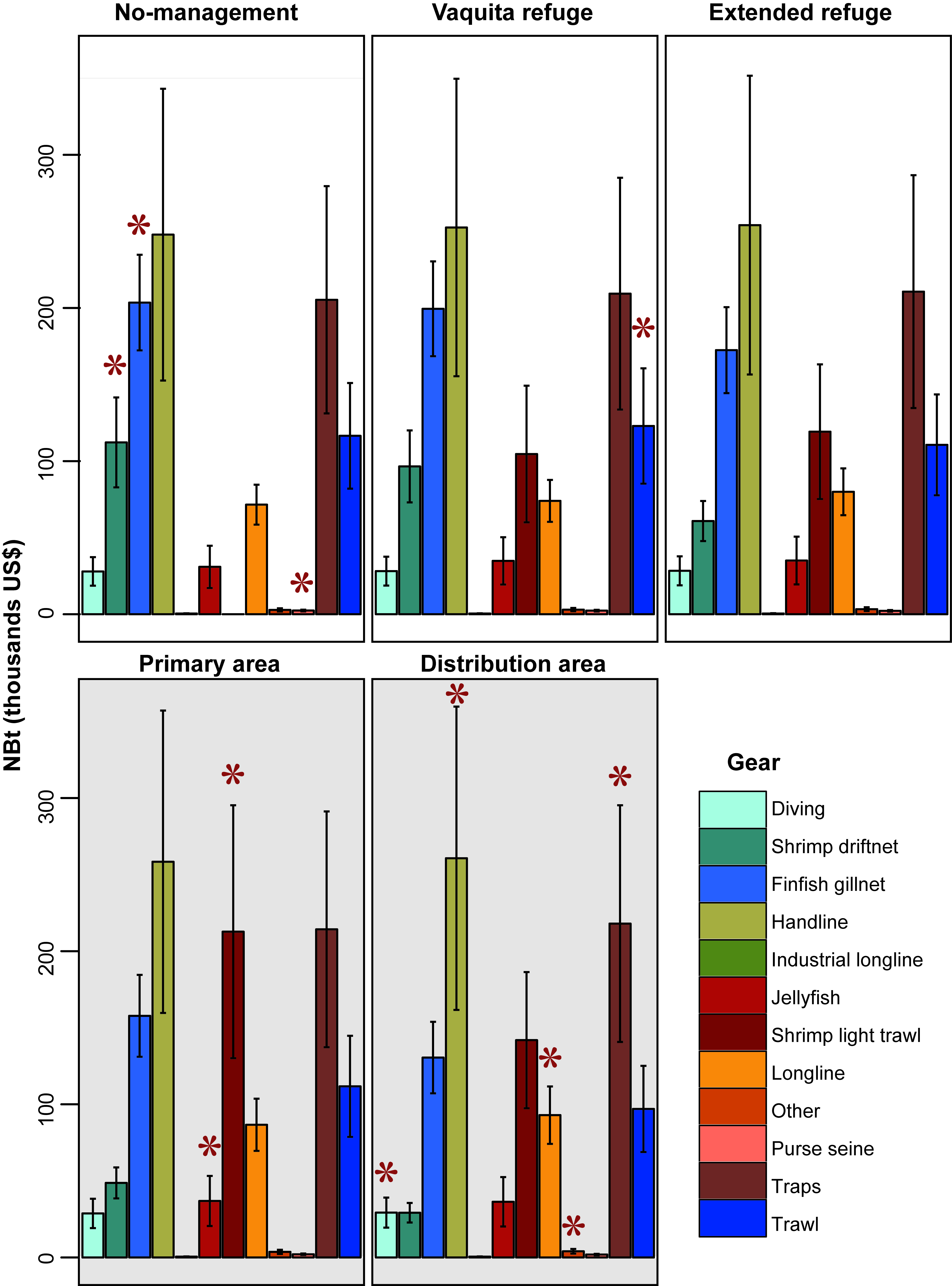

Supplement: Figure S2 — Undiscounted net benefit for each gear and management scenario. Values are averages for the last 5 years of the 30-year simulations (± SE). (*) indicates the scenario with the highest value for each gear. See text for calculation of net benefit. (TIF) [file pone.0042917.s002.tif]

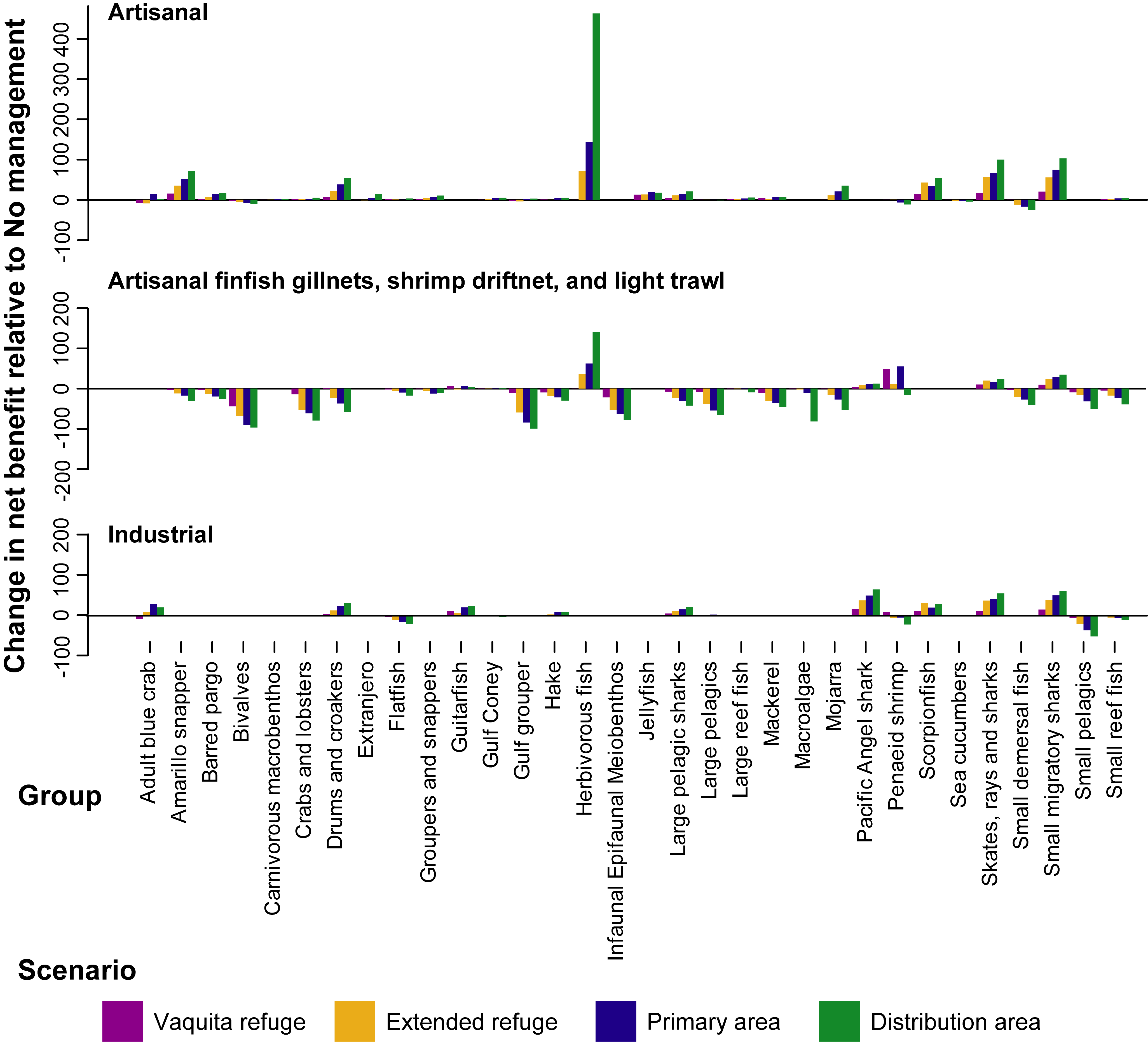

Supplement: Figure S3 — Percent change in undiscounted net benefit deriving from harvest of selected functional groups across scenarios, relative to No management. Net benefit is average for the last 5 years of the 30-year simulations. (TIF) [file pone.0042917.s003.tif]

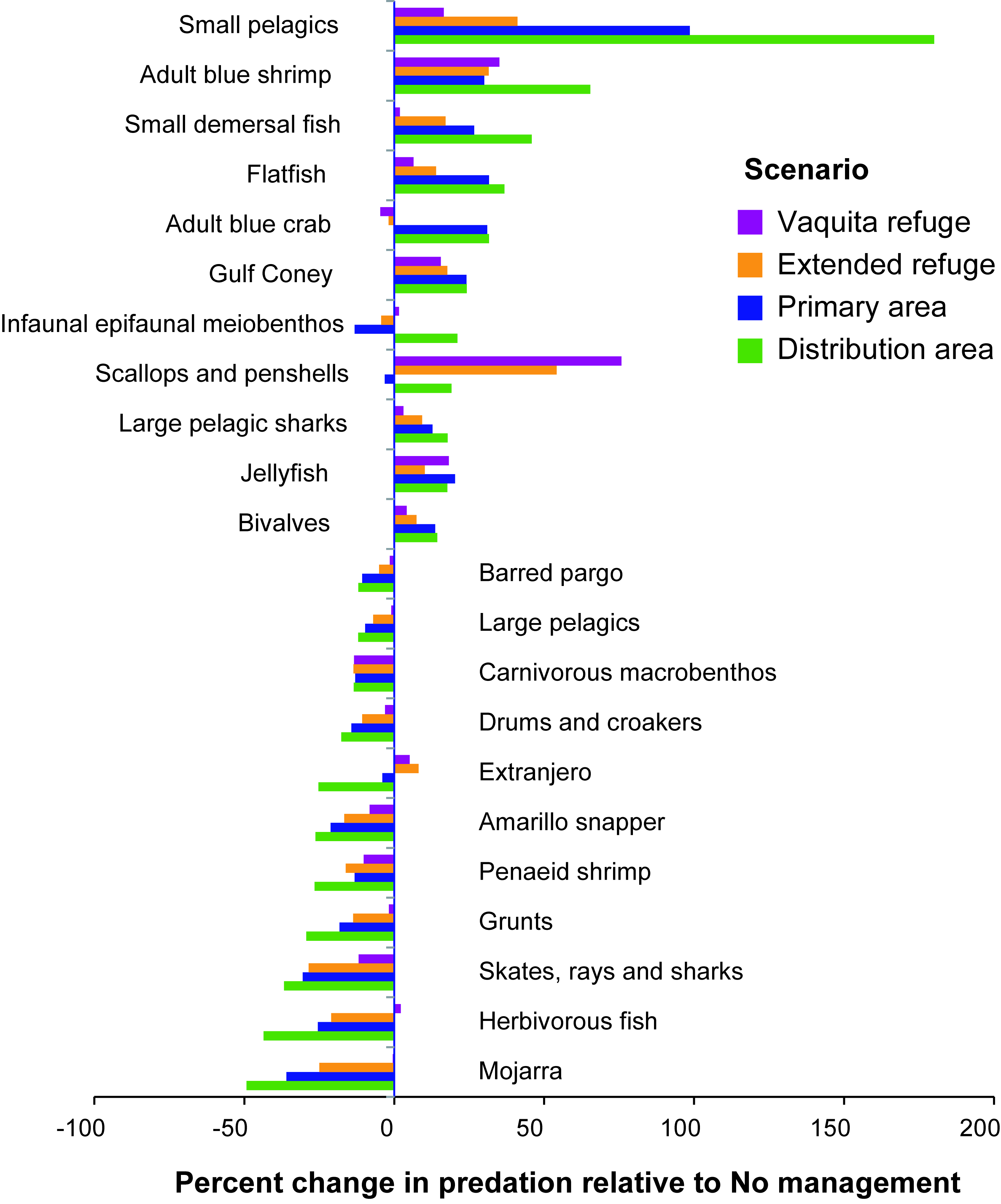

Supplement: Figure S4 — Percent increase in predation for selected target groups across scenarios relative to No management, in the last year of the 30-year simulation. (TIF) [file pone.0042917.s004.tif]
