# Supplementary material for: Exploring Trade-Offs between Fisheries and Conservation of the Vaquita Porpoise (Phocoena sinus) Using an Atlantis Ecosystem Model
Source: PLoS One. 2012 Aug 15;7(8):e42917. doi: 10.1371/journal.pone.0042917 (PMC3419746; doi:10.1371/journal.pone.0042917)
Supplement: Text S1 — Management actions for vaquita conservation. (DOCX) [file pone.0042917.s005.docx]

**Text S1. Management actions for vaquita conservation.**

The Mexican government established the Upper Gulf of California and Colorado River Delta Biosphere Reserve (‘Biosphere reserve’) in 1993 to protect the vaquita and other endemic species [1]. Gillnets were banned from the core area of the Biosphere Reserve (Figure 1), near the mouth of the Colorado River. In conjunction, gillnets for Totoaba (*Cynoscion macdonaldi*) of mesh size > 12 in, which had a high vaquita bycatch, were banned throughout the Gulf [2]. A survey of vaquita abundance and distribution [3] found that the Biosphere Reserve’s boundaries did not correspond with the distribution of vaquita and that sightings were concentrated outside of the reserve’s core area.

In 2002, in response to recommendations by the International Committee for the Recovery of Vaquita (CIRVA for its Spanish acronym; [4], the government banned shrimp trawls and most large-mesh gillnets within the Biosphere Reserve; protests by affected communities led Congress to quickly derogate the law [5]. A Vaquita refuge (~1271 km^2^) was eventually established in 2005 (Figure 1; [6]. The refuge contained within its borders the positions of approximately 80% of verified vaquita sightings [7]. Gillnets of > 6 inches in mesh were banned in the refuge, trawl effort was reduced and a no-fish zone was declared within a ~270 km^2^ polygon where vaquita sightings were concentrated. A new management program for the Biosphere Reserve banned gillnets and trawls from the portion of the Vaquita refuge within the reserve [8]. Initially, there was little enforcement and low compliance with these regulations [7,9].

Following the vaquita’s listing as Critically Endangered in the IUCN Red List of Threatened Species and in response to sustained pressure from the scientific and international community [7,10], a comprehensive species recovery program was published in 2008 (PACE for its Spanish acronym; [11]). PACE established the goal of removal of gillnets and trawls from vaquita refuge by 2010 and from the entire vaquita distribution area (~ 8432 km^2^; Figure 1) by 2012 through buyouts, where fishermen turned in their boats, gears and permits in return for a payment that could be invested in an alternative business; switchouts, where fishermen could continue fishing for shrimp but with an alternate gear that eliminates vaquita mortality and also receive a monetary compensation; and rent-outs, where fishermen where paid to stop fishing in the vaquita refuge but retained their boats, permits and gears [12]. PACE invested 35 million pesos in 2007, 153 million pesos in 2008, and had distributed 69 million pesos by the third trimester of 2010; [12,13]. Participation in the buy-out option resulted in 181 boats retiring from the fishery; but enrollment in this option decreased over time [14].

Continued work by the Mexican Fisheries Secretariat seeks to develop gears for the shrimp fishery that eliminate vaquita mortality [15]; fishers are paid to test the prototypes as part of the switchout program. The government has also stepped up patrols to verify permits and gear restrictions within the vaquita’s limited ~4000 km2 distribution area (S. Perez-Valencia, CEDO Intercultural. Puerto Peñasco, Sonora).

**References**

1. DOF (1993) Decreto por el que se declara área natural protegida con el carácter de Reserva de la Biosfera, la región conocida como Alto Golfo de California y Delta del Rio Colorado, ubicada en aguas del Golfo de California y los municipios de Mexicali, B.C., del Puerto Peñasco y San Luis Rio Colorado, Sonora. Diario Oficial de la Federación. June 10, 1993. Available: http://www.conanp.gob.mx/sig/decretos/reservas/Altogolfo.pdf. Accessed 2012 July 17.

2. DOF (1994) Norma Oficial Mexicana NOM-024-SEMARNAT-1993, por la que se establecen medidas para la protección de las especies de totoaba y vaquita en aguas de jurisdicción federal del Golfo California. Secretaría del Medio Ambiente y Recursos Naturales. Diario Oficial de la Federación. May 30, 1994. Available: http://www.semarnat.gob.mx/leyesynormas/normas. Accessed 2012 July 17.

3. Jaramillo-Legorreta A, Rojas-Bracho L, Gerrodette T (1999) A new abundance estimate for vaquitas: first step for recovery. Mar Mammal Sci 15: 957–973.

4. CIRVA (1997) Report of the first meeting of the International Committee for the recovery of the Vaquita (CIRVA). Ensenada, Baja California, México. 23 p. Available: http://www.vivavaquita.org/PDF/International%28CIRVA%2904.pdf. Accessed 2012 July 17.

5. Turk-Boyer PJ (2006) CEDO. In: Felger RS, Broyles B, editors. Dry Borders: Great Natural Reserves of the Sonoran Desert. Salt Lake City, Utah: University of Utah Press. pp. 548–559.

6. DOF (2005) ACUERDO mediante el cual se establece el área de refugio para la protección de la vaquita (*Phocoena sinus*). Secretaría del Medio Ambiente y Recursos Naturales. Diario Oficial de la Federación. Sept 8, 2005. Available: http://www.ine.gob.mx/con-eco-vaquita/419-vaquita-acuerdo. Accessed 2012 July 17.

7. Rojas-Bracho L, Reeves RR, Jaramillo-Legorreta A (2006) Conservation of the vaquita *Phocoena sinus*. Mammal Rev 36: 179–216.

8. CONANP (2007) Programa de conservación y manejo de la Reserva de la Biosfera Alto Golfo de California y Delta del Río Colorado. México, D.F.: Comisión Nacional de Areas Naturales Protegidas. 319 p.

9. Gerrodette T, Rojas-Bracho L (2011) Estimating the success of protected areas for the vaquita, *Phocoena sinus*. Mar Mammal Sci 27: E101–E125. doi:10.1111/j.1748-7692.2010.00449.x.

10. Jaramillo-Legorreta A, Rojas-Bracho L, Brownell RL, Read AJ, Reeves RR, et al. (2007) Saving the vaquita: Immediate action, not more data. Conserv Biol 21: 1653–1655.

11. SEMARNAT (2008) Programa de Acción para la Conservación de la Especie: Vaquita (*Phocoena sinus*). Estrategia integral para el manejo sustentable de los recursos marinos y costeros del Alto Golfo de California. Mexico, D.F. Secretaría del Medio Ambiente y Recursos Naturales. 106 p. Available:http://www.conanp.gob.mx/pdf_especies/PACEvaquita.pdf.

12. SEMARNAT, CONANP (2010) Lineamientos para el otorgamiento de apoyos del Programa de Acción para la Conservación de la Especie: Vaquita (*Phocoena sinus*). Ejercicio fiscal 2010. Available: http://www.conanp.gob.mx/contenido/pdf/LINEAMIENTOS%20PACE%20VAQUIITA%2020100001__.pdf. Accessed 2012 July 17.

13. Ruiz-López DM (2009) Diagnóstico socioéconomico y evaluación de una estrategia de compensación al sector pesquero de El Golfo de Santa Clara, Sonora [M.S. Thesis. Recursos Naturales y Medio Ambiente]. Guasave, Sinaloa: Centro Interdisciplinario de Investigación para el Desarrollo Integral Regional. Instituto Politécnico Nacional. Available: http://itzamna.bnct.ipn.mx:8080/dspace/handle/123456789/8211. Accessed 2012 July 17.

14. Avila-Forcada S, Martínez-Cruz AL, Muñoz-Piña C (2012) Conservation of vaquita marina in the Northern Gulf of California. Mar Policy 36: 613–622. doi:10.1016/j.marpol.2011.10.012.

15. INAPESCA, NMFS (2012) Fishing technology research collaboration: Efficiency and selectivity of two trawl nets design to fish blue shrimp (*Litopenaeus stylirostris*) in the artisanal shrimp fishery of the Upper Gulf of California. 9 p.
