# Supplementary material for: Exploring Trade-Offs between Fisheries and Conservation of the Vaquita Porpoise (Phocoena sinus) Using an Atlantis Ecosystem Model
Source: PLoS One. 2012 Aug 15;7(8):e42917. doi: 10.1371/journal.pone.0042917 (PMC3419746; doi:10.1371/journal.pone.0042917)
Supplement: Text S2 — Historical vaquita abundance trajectory (1993–2008). (DOCX) [file pone.0042917.s006.docx]

**Text S2. Historical vaquita abundance trajectory (1993-2008).**

In order to calibrate dynamic parameters in the Atlantis ecosystem simulation model for the Northern Gulf of California, it was necessary to compare the predicted dynamics of all ecosystem components with available historical time series of catch and relative abundance. We chose as a starting point for the historical model the year 1985 because we had the most comprehensive record of time series catch and relative abundance data from that point. In addition, it is the earliest available year provided by the regional ocean modeling system (ROMS) model. The development of the historic 1985 model for the Northern Gulf, the development of time series data from literature references, monitoring, and local environmental knowledge [1] and the calibration process and fits to data are detailed in Ainsworth et al. [2].

To compare the historical vaquita abundance trajectory modeled by Atlantis to the 1993-2008 trajectory derived through a Bayesian population model [3], we recreated a 1993 model based on the historical 1985 model. For species other than vaquita, we used the corresponding historical catch data and estimated abundance based on a linear interpolation between 1985 and 2008 to obtain a 1993 starting point. We also assumed stationarity in feeding behavior and life history parameters of organisms [4]. We initialized simulations using 664 vaquitas, the median estimated vaquita abundance for 1993 [3]. We applied a variable annual mortality rate, using the estimates from Gerrodette and Rojas-Bracho [3] that indicate that the fraction of the population taken annually as bycatch increased from 0.09 year^-1^ in 1993 to 0.15 year^-1^ in 2007. We then drove the 1993 model forward 16 years under historical fishing patterns [2] and oceanography described by the ROMS model.

The resulting vaquita abundance trajectory for 1993-2008 is within the 95% confidence intervals for published abundance estimates based on transect and acoustic survey data [5–7]. The Atlantis model abundance trajectory follows the Gerrodette and Rojas-Bracho [3] model estimates for the first 5 years of the simulation, but the population then decreases relative to this estimate. By 2008, Atlantis predicts 100 fewer vaquitas (114 vs 215). For 2008, the population size predicted by Atlantis model is closer to the 76 vaquitas estimated by Jaramillo-Legorreta [8] for 2008.


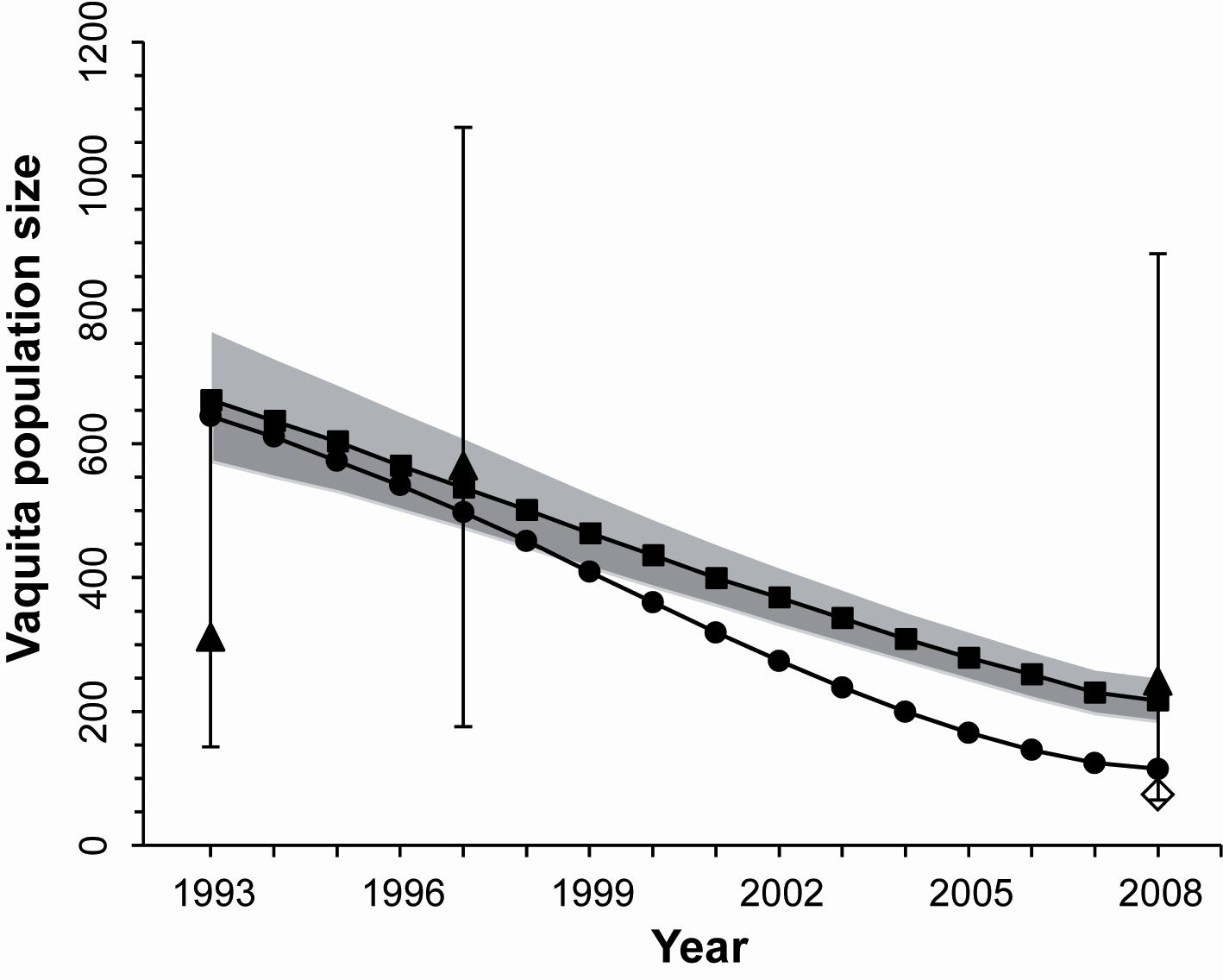


In this figure, the circles represent the Atlantis model abundance trajectory for 1993-2008, the squares correspond to the medians of the posterior distribution from Gerrodette and Rojas-Bracho [3] and the grey shading is the 50% and 95% of probability density modeled by Gerrodette and Rojas-Bracho [3], Figure 7A; the open diamond is the model estimate of Jaramillo-Legorreta [8] for 2008; and the triangles are 1993, 1997 and 2008 abundance estimates:

| **Year** | **Estimate** | **(95% CI)** | **Reference** |
| --- | --- | --- | --- |
| 1993 | 311 | (147,653) | Barlow et al. 1997; Jaramillo-Legorreta et al. 1999 |
| 1997 | 567 | (177,1073) | Jaramillo-Legorreta et al. 1999. Combined estimate |
| 2008 | 245 | (68–884) | Gerrodette et al. 2011. Total |

**References**

1. Ainsworth CH (2011) Quantifying species abundance trends in the Northern Gulf of California using Local Ecological Knowledge. Mar Coast Fish Dyn Mgmt Ecosys Sci 3: 190–218.

2. Ainsworth C, Kaplan IC, Levin PS, Cudney-Bueno R, Fulton EA, et al. (2011) Atlantis model development for the Northern Gulf of California. NOAA Technical Memorandum NMFS-NWFSC-110. Department of Commerce. National Oceanic and Atmospheric Administration. National Marine Fisheries Service. Seattle, WA, USA. 293 p. Available:http://www.nwfsc.noaa.gov/assets/25/7784_08012011_125850_AtlantisModelTM110WebFinal.pdf. Accessed 2012 July 17.

3. Gerrodette T, Rojas-Bracho L (2011) Estimating the success of protected areas for the vaquita, *Phocoena sinus*. Mar Mammal Sci 27: E101–E125. doi:10.1111/j.1748-7692.2010.00449.x.

4. Ainsworth CH, Varkey DA, Pitcher TJ (2008) Ecosystem simulations supporting ecosystem-based fisheries management in the Coral Triangle, Indonesia. Ecol Model 214: 361–374. doi:10.1016/j.ecolmodel.2008.02.039.

5. Barlow J, Gerrodette T, Silber G (1997) First estimates of vaquita abundance. Mar Mammal Sci 13: 44–55. doi:10.1111/j.1748-7692.1997.tb00611.x.

6. Gerrodette T, Taylor BL, Swift R, Rankin S, Jaramillo-Legorreta AM, et al. (2011) A combined visual and acoustic estimate of 2008 abundance, and change in abundance since 1997, for the vaquita, *Phocoena sinus*). Mar Mammal Sci 27: E97–E100. doi:10.1111/j.1748-7692.2010.00438.x.

7. Jaramillo-Legorreta A, Rojas-Bracho L, Gerrodette T (1999) A new abundance estimate for vaquitas: first step for recovery. Mar Mammal Sci 15: 957–973.

8. Jaramillo-Legorreta A (2008) Estatus actual de una especie en peligro de extinción, la vaquita (*Phocoena sinus*): Una aproximación poblacional con métodos acústicos y bayesianos [PhD dissertation]. Ensenada, B.C.: Universidad Autonoma de Baja California, Facultad de Ciencias Marinas. 129 p.
