# Supplementary material for: Exploring Trade-Offs between Fisheries and Conservation of the Vaquita Porpoise (Phocoena sinus) Using an Atlantis Ecosystem Model
Source: PLoS One. 2012 Aug 15;7(8):e42917. doi: 10.1371/journal.pone.0042917 (PMC3419746; doi:10.1371/journal.pone.0042917)
Supplement: Table S1 — Vaquita diet based on averaged values from the ecosystem models developed by Morales-Zárate [1] and Lozano [2] ; based on data analyzed by [3], [4] . Diet for juveniles was assumed similar to adults. Values are proportion of total diet for each functional group used at the start of the simulations, realized diets will vary through time and space based on prey availability. (DOCX) [file pone.0042917.s007.docx]

| **Functional group** | **Proportion** |
| --- | --- |
| Squid | >0.0001 |
| Crabs and lobsters | >0.0001 |
| Carnivorous macrobenthos | >0.0001 |
| Groupers and snappers | 0.001 |
| Hake | 0.002 |
| Totoaba | 0.015 |
| Small demersal fish | 0.017 |
| Herbivorous fish | 0.020 |
| Flatfish | 0.023 |
| Lanternfish and deep | 0.035 |
| Scorpionfish | 0.048 |
| Mojarra | 0.073 |
| Drums and croakers | 0.094 |
| Grunts | 0.112 |
| Small pelagics | 0.559 |

1. Morales-Zarate MV, Arreguin-Sanchez F, Lopez-Martinez J, Lluch-Cota SE (2004) Ecosystem trophic structure and energy flux in the Northern Gulf of California, Mexico. Ecol Model 174: 331–345.

2. Lozano H (2006) Historical ecosystem modelling of the Upper Gulf of California (Mexico): Following 50 years of change. [PhD dissertation]. Vancouver, British Columbia: The University of British Columbia, The Faculty of Graduate Studies. 266 p.

3. Pérez-Cortés Moreno H, Silber GK, Villa-Ramírez B (1996) Contribución al conocimiento de la alimentación de la vaquita *Phocoena sinus*. INP-SEMARNAP Ciencia Pesquera: 66–72.

4. Vidal O, Brownell Jr. RL, Findley LT (1999) Vaquita Phocoena sinus Norris and McFarland, 1958. In: Ridgway SH, Harrison RJ, editors. The second book of dolphins and the porpoises. Handbook of Marine Mammals: Volume 6. San Diego, CA Tempe AZ: Academic Press. pp. 357–378.
