# Supplementary material for: Exploring Trade-Offs between Fisheries and Conservation of the Vaquita Porpoise (Phocoena sinus) Using an Atlantis Ecosystem Model
Source: PLoS One. 2012 Aug 15;7(8):e42917. doi: 10.1371/journal.pone.0042917 (PMC3419746; doi:10.1371/journal.pone.0042917)
Supplement: Table S2 — Catch by functional group used as baseline in the No management scenario. Catches are the average of the 2000–2007 model catch series from Ainsworth et al. [1] summed for all fleets and modified as described in Ainsworth et al. [2]. Vaquita mortality was set at 0.15 year-1, the median estimate prior to 2007 [3]. Since the publication of Ainsworth et al. [1], the model has been simplified to only include catch for a generic Penaeid shrimp group rather than for separate shrimp groups. Ainsworth et al. [1] provide species composition for each functional group. (DOCX) [file pone.0042917.s008.docx]

| **Functional group** | **Catch (t)** | **Functional group** | **Catch (t)** |
| --- | --- | --- | --- |
| Gulf Coney | 1230.84 | Vaquita | 0.82 |
| Extranjero | 2743.55 | Pinnipeds | 211.98 |
| Leopard grouper | 560.96 | Oceanic sea turtles | 47.68 |
| Gulf grouper | 186.94 | Reef associated turtles | 16.79 |
| Amarillo snapper | 292.77 | Sea birds | 34.67 |
| Barred pargo | 346.70 | Benthic bacteria | 0.00 |
| Groupers & snappers | 20209.43 | Scallops & pen shells | 146.27 |
| Drums & croakers | 41676.80 | Penaeid shrimp | 14249.19 |
| Grunts | 5313.45 | Sea cucumbers | 300.02 |
| Herbivorous fish | 4171.02 | Sessile invertebrates | 37.82 |
| Lg. reef fish | 6958.88 | Crabs & lobsters | 21748.72 |
| Sm. reef fish | 5969.91 | Herbivorous echinoderms | 0.23 |
| Sm. demersal fish | 15568.09 | Carnivorous macrobenthos | 1549.31 |
| Pacific Angel shark | 401.51 | Infaunal epifaunal meiobenthos | 39.48 |
| Sm. migratory sharks | 15763.16 | Bivalves | 1791.27 |
| Lg. pelagic sharks | 1704.44 | Snails | 930.65 |
| Guitarfish | 17438.68 | Adult blue crab | 3340.10 |
| Skates, rays & sharks | 25923.41 | Juv blue crab | 0.00 |
| Flatfish | 4458.66 | Labile detritus | 0.00 |
| Mojarra | 2973.86 | Refractory detritus | 0.00 |
| Scorpionfish | 3246.59 | Macroalgae | 323.57 |
| Lanternfish & deep | 1178.87 | Pelagic bacteria | 0.00 |
| Totoaba | 39.80 | Lg. phytoplankton | 0.00 |
| Lg. pelagics | 2186.54 | Sm. phytoplankton | 0.00 |
| Mackerel | 644.26 | Seagrass | 0.00 |
| Hake | 211.33 | Jellyfish | 1676.79 |
| Sm. pelagics | 47289.00 | Lg. zooplankton | 0.00 |
| Mysticeti | 389.43 | Sm. zooplankton | 0.00 |
| Odontocetae | 581.98 | Microphytobenthos | 0.00 |
| Orca | 0.00 | Carrion detritus | 0.00 |

1. Ainsworth C, Kaplan IC, Levin PS, Cudney-Bueno R, Fulton EA, et al. (2011) Atlantis model development for the Northern Gulf of California. NOAA Technical Memorandum NMFS-NWFSC-110. Department of Commerce. National Oceanic and Atmospheric Administration. National Marine Fisheries Service. Seattle, WA, USA. 293 p. Available: http://www.nwfsc.noaa.gov/assets/25/7784_08012011_125850_AtlantisModelTM110WebFinal.pd Accessed 2012 July 17.

2. Ainsworth CH, Morzaria-Luna H, Kaplan IC, Levin PS, Fulton EA (2012) Full compliance with harvest regulations yields ecological benefits: Northern Gulf of California case study. J Appl Ecol 49: 63–72. doi:10.1111/j.1365-2664.2011.02064.x.

3. Gerrodette T, Rojas-Bracho L (2011) Estimating the success of protected areas for the vaquita, *Phocoena sinus*. Mar Mammal Sci 27: E101–E125. doi:10.1111/j.1748-7692.2010.00449.x.
