# Supplementary material for: Exploring Trade-Offs between Fisheries and Conservation of the Vaquita Porpoise (Phocoena sinus) Using an Atlantis Ecosystem Model
Source: PLoS One. 2012 Aug 15;7(8):e42917. doi: 10.1371/journal.pone.0042917 (PMC3419746; doi:10.1371/journal.pone.0042917)
Supplement: Table S3 — Fishery fleets in the Atlantis Northern Gulf of California model. Fleets are defined based on gears, targets, bycatch, home ports and fishery utilization areas. Table indicates ports, functional groups targeted out of 63 in the model and gears. See Ainsworth et al. [1] and Ainsworth [2] for more details on fleets and functional groups used. (DOCX) [file pone.0042917.s009.docx]

| **#** | **Fleet name** | **Base ports** | **# groups** | **Gear** | **Main groups targeted** |
| --- | --- | --- | --- | --- | --- |
| 1 | Industrial shrimp trawl | Guaymas | 39 | Trawl net | Shrimp |
| 2 | Industrial shrimp trawl | Puerto Peñasco | 39 | Trawl net | Shrimp |
| 3 | Shrimp driftnet  Upper Gulf | Desemboque, Golfo de Santa Clara, Puerto Peñasco, San Felipe, San Jorge, San Luis | 7 | Gill net | Shrimp |
| 4 | Shrimp driftnet Kino | Bahia Kino | 6 | Gill net | Shrimp |
| 5 | Finfish trawl | All | 12 | Trawl net | Finfish |
| 6 | Offshore demersal gillnet | All | 24 | Gillnet | Small migratory sharks |
| 7 | Inshore demersal shark fleet | All | 5 | Longline and gillnet | Sharks, Angel Shark, migratory Sharks, guitarfish |
| 8 | Inshore pelagic shark fleet | All | 1 | Longline and gillnet | Large pelagic sharks |
| 9 | Inshore demersal gillnet | All | 16 | Gillnet | Guitarfish, skates, flatfish, scorpionfish |
| 10 | Inshore gillnet Curvina golfina | Golfo de Santa Clara, Puerto Peñasco, San Felipe | 10 | Gillnet | *Cynoscion othonopterus* |
| 11 | Inshore gillnet other | All | 13 | Gillnet | Mullets, Sea turtles |
| 12 | Offshore pelagic gillnet | All | 13 | Gillnet | Sierra, Jacks |
| 13 | Industrial pelagic longline (medium boats) | All | 4 | Longline | Sharks, tuna, jacks & finfish |
| 14 | Longlines (small skiffs, demersal) | All | 14 | Longline | Gulf coney, extranjero, mustelids |
| 15 | Longlines (small skiffs, pelagic) | All | 7 | Longline | Sharks, rays, drums, croakers, groupers, snappers |
| 16 | Demersal handline | All | 17 | Handline | Lutjanids, Serranids, Hemulidae, triggerfish |
| 17 | Pelagic handline | All | 13 | Handline | Scombridae, Carrangidae |
| 18 | Blue crab traps | Bahia Kino, Desemboque Seris, Punta Chueca | 1 | Traps | Blue crab |

# Table S3 (continued)

|  | **Fleet name** | **Base ports** | **# groups** | **Gear** | **Main groups targeted** |
| --- | --- | --- | --- | --- | --- |
| 19 | Blue crab traps | Ports North of Desemboque | 1 | Traps | Blue crab |
| 20 | Octopus traps | All | 1 | Traps | Octopus |
| 21 | Fish and lobster traps | All | 9 | Traps | Extranjero, groupers and snappers, large reef fish |
| 22 | Compressor diving | All | 13 | Hooks, harpoons, manual | Clams, oysters, cucumbers, snails, lobster, sea turtles |
| 23 | Octopus compressor diving | All | 1 | Hooks, chemical | Octopus |
| 24 | Pen shell compressor diving | All | 1 | Manual | Pen shells |
| 25 | Sea cucumber compressor diving | All | 1 | Manual | Sea cucumber |
| 26 | Geoduck compressor diving | All | 1 | Pressure hose | Infaunal / epifaunal meiobenthos, scallops and pen shells, carnivorous macrobenthos, bivalves |
| 27 | Jellyfish hand net | All | 1 | Handnet | Jellyfish |
| 28 | Recreational fishing | All | 14 | Fishing pole | Large pelagic, medium pelagic, reef fish |
| 29 | Tuna purse seine | All | 6 | Purse seine | Tuna |
| 30 | Small pelagic purse seine | All | 4 | Purse seine | Small pelagic fish |
| 31 | Macroalgae | All | 1 | Manual | Macroalgae |
| 32 | Other gears | All | 9 | Cast net, harpoons, beach seine, dynamite | Finfish |

1. Ainsworth C, Kaplan IC, Levin PS, Cudney-Bueno R, Fulton EA, et al. (2011) Atlantis model development for the Northern Gulf of California. NOAA Technical Memorandum NMFS-NWFSC-110. Department of Commerce. National Oceanic and Atmospheric Administration. National Marine Fisheries Service. Seattle, WA, USA. 293 p. Available:http://www.nwfsc.noaa.gov/assets/25/7784_08012011_125850_AtlantisModelTM110WebFinal.pdf. Accessed 2012 July 17.

2. Ainsworth CH, Morzaria-Luna H, Kaplan IC, Levin PS, Fulton EA (2012) Full compliance with harvest regulations yields ecological benefits: Northern Gulf of California case study. J Appl Ecol 49: 63–72. doi:10.1111/j.1365-2664.2011.02064.x.
