# Supplementary material for: Exploring Trade-Offs between Fisheries and Conservation of the Vaquita Porpoise (Phocoena sinus) Using an Atlantis Ecosystem Model
Source: PLoS One. 2012 Aug 15;7(8):e42917. doi: 10.1371/journal.pone.0042917 (PMC3419746; doi:10.1371/journal.pone.0042917)
Supplement: Table S4 — Catch per fleet used as a baseline. Asterisk indicates group is bycatch; fleet name is in bold. Vaquita catch was updated considering a mortality rate of 0.15 year-1. Bycatch composition for the Upper Gulf shrimp driftnet fleet was updated using more monitoring data [1]. Otherwise catch per fleet values are unmodified from Ainsworth et al. [2], [3]. (DOCX) [file pone.0042917.s010.docx]

| **Fleet/ group caught** | **Catch (t)** | **Fleet/ group caught** | **Catch (t)** | **Fleet/ group caught** | **Catch (t)** |
| --- | --- | --- | --- | --- | --- |
| **Ind. shrimp trawl Guaymas** | | **Ind. shrimp trawl Peñasco (cont.)** | | **Shrimp driftnet Upper Gulf (cont.)** | |
| Crabs & lobsters | 18845.33 | Scorpionfish | 2151.01 | Bivalves | 25.43 |
| Penaeid shrimp | 1721.94 | Groupers & snappers | 1831.16 | Scorpionfish | 25.40 |
| Grunts | 1612.36 | Guitarfish | 1727.90 | Jellyfish | 20.89 |
| Groupers & snappers | 1438.01 | Sm. reef fish | 1335.18 | Sm. migratory sharks | 17.16 |
| Sm. demersal fish | 1236.19 | Lg. reef fish | 930.52 | Groupers & snappers | 16.98 |
| Drums & croakers | 947.95 | Lg. Pelagics | 260.12 | Herbivorous fish | 16.95 |
| Sm. reef fish | 740.13 | Adult blue crab | 208.36 | Macroalgae | 16.94 |
| Carnivorous macrobenthos | 606.28 | Lanternfish & deep | 147.74 | Guitarfish | 8.55 |
| Extranjero | 429.72 | Mysticeti | 138.69 | Lg. pelagic sharks | 8.52 |
| Lg. reef fish | 408.23 | Odontocetae | 138.69 | Mojarra | 8.50 |
| Sm. pelagics | 313.34 | Carnivorous macrobenthos | 90.18 | Gulf Coney | 8.47 |
| Scorpionfish | 294.12 | Sm. migratory sharks | 42.33 | Pacific Angel shark | 8.47 |
| Lg. pelagics | 276.89 | Snails | 26.53 | Sea cucumbers | 8.47 |
| Mysticeti | 230.38 | Extranjero | 16.13 | Totoaba | 8.46 |
| Odontocetae | 230.38 | Totoaba | 11.13 | Pinnipeds | 5.72 |
| Snails | 178.38 | Flatfish | 8.23 | Odontocetae | 5.43 |
| Skates, rays & sharks | 88.51 | Mojarra | 5.49 | Sea birds | 0.94 |
| Lanternfish & deep | 71.80 | Inf. epi. meiobenthos | 3.87 | Vaquita | 0.39 |
| Adult blue crab | 56.79 | Lg. pelagic sharks | 3.15 | Mysticeti | 0.24 |
| Sm. migratory sharks | 51.37 | Sessile invertebrates | 3.12 | Oceanic sea turtles | 0.08 |
| Mackerel | 34.52 | Sea cucumbers | 1.96 | Reef associated turtles | 0.04 |
| Sea cucumbers | 13.17 | Pacific Angel shark | 0.74 | **Shrimp driftnet Kino** |  |
| Barred pargo | 8.29 | Scallops & pen shells | 0.20 | Penaeid shrimp | 172.67 |
| Herbivorous fish | 7.73 | Bivalves | 0.20 | Sm. demersal fish | 39.58 |
| Amarillo snapper | 4.14 | Herbivorous echinoderms | 0.03 | Sm. migratory sharks | 37.64 |
| Guitarfish | 3.44 | **Shrimp driftnet Upper Gulf** | | Sm. reef fish | 32.49 |
| Flatfish | 2.24 | Penaeid shrimp | 866.06 | Drums & croakers | 22.90 |
| Mojarra | 1.50 | Sm. demersal fish | 110.42 | Lg. reef fish | 12.07 |
| Scallops & penshells | 1.37 | Flatfish | 110.08 | Lg. pelagics | 12.07 |
| Bivalves | 1.37 | Drums & croakers | 93.33 | Mackerel | 4.31 |
| Inf. epi. meiobenthos | 1.05 | Crabs & lobsters | 84.67 | Mojarra | 3.41 |
| Lg. pelagic sharks | 0.86 | Snails | 67.72 | Herbivorous fish | 3.24 |
| Sessile invertebrates | 0.85 | Skates, rays & sharks | 59.33 | Pinnipeds | 1.21 |
| Gulf Coney | 0.83 | Carnivorous macrobenthos | 42.41 | Odontocetae | 1.15 |
| Pacific Angel shark | 0.20 | Lg. Pelagics | 42.35 | Sea birds | 0.20 |
| Herbivorous echinoderms | 0.20 | Inf. epi. meiobenthos | 34.56 | Mysticeti | 0.05 |
| **Ind. shrimp trawl Peñasco** | | Sm. reef fish | 34.05 | Oceanic sea turtles | 0.02 |
| Penaeid shrimp | 11486.40 | Grunts | 33.93 | Reef associated turtles | 0.01 |
| Sm. demersal fish | 10477.21 | Sessile invertebrates | 33.86 | **Finfish trawl** |  |
| Drums & croakers | 9395.76 | Sm. Pelagics | 25.64 | Skates, rays & sharks | 10348.37 |
| Skates, rays & sharks | 8285.87 | Lg. reef fish | 25.47 | Guitarfish | 8432.90 |
| Crabs & lobsters | 2803.19 | Adult blue crab | 25.46 | Drums & croakers | 7700.92 |
| Grunts | 2464.11 | Mackerel | 25.43 | Lanternfish & deep | 959.33 |

**Table S4 (continued)**

| **Fleet/ group caught** | **Catch (t)** | **Fleet/ group caught** | **Catch (t)** | **Fleet/ group caught** | **Catch (t)** |
| --- | --- | --- | --- | --- | --- |
| **Finfish trawl (cont.)** |  | **Inshore demersal shark fleet (cont.)** | | **Inshore gillnet other (cont.)** | |
| Sm. reef fish | 719.74 | Vaquita | 0.03 | Sea birds | 8.40 |
| Flatfish | 446.16 | **Inshore pelagic shark fleet** | | Lg. reef fish | 6.57 |
| Scorpionfish | 400.73 | Lg. pelagic sharks | 21.31 | Carnivorous macrobenthos | 2.55 |
| Sm. migratory sharks | 310.23 | **Inshore demersal gillnet** | | Mysticeti | 2.12 |
| Gulf Coney | 12.31 | Flatfish | 1901.36 | Reef associated turtles | 0.37 |
| Mysticeti | 11.59 | Sm. demersal fish | 621.00 | Vaquita | 0.11 |
| Odontocetae | 11.59 | Mojarra | 485.06 | **Offshore pelagic gillnet** |  |
| Totoaba | 1.41 | Grunts | 114.12 | Drums & croakers | 1498.54 |
| **Offshore demersal gillnet** | | Adult blue crab | 67.77 | Mackerel | 403.72 |
| Sm. migratory sharks | 13307.78 | Sm. reef fish | 60.92 | Lg. pelagic sharks | 255.54 |
| Guitarfish | 7186.66 | Sm. Pelagics | 47.29 | Amarillo snapper | 234.66 |
| Skates, rays & sharks | 3258.82 | Lg. reef fish | 45.14 | Sm. reef fish | 225.16 |
| Groupers & snappers | 3249.82 | Pinnipeds | 11.92 | Lg. pelagics | 92.10 |
| Flatfish | 1901.36 | Odontocetae | 11.32 | Pinnipeds | 9.69 |
| Drums & croakers | 1498.54 | Sea birds | 1.95 | Odontocetae | 9.20 |
| Sm. demersal fish | 621.00 | Totoaba | 1.56 | Lg. reef fish | 6.57 |
| Pacific Angel shark | 384.06 | Mysticeti | 0.49 | Sea birds | 1.58 |
| Mackerel | 172.08 | Scorpionfish | 0.35 | Mysticeti | 0.40 |
| Lg. pelagic sharks | 127.77 | Reef associated turtles | 0.09 | Oceanic sea turtles | 0.13 |
| Pinnipeds | 114.67 | Leopard grouper | 0.06 | Vaquita | 0.02 |
| Grunts | 114.12 | Vaquita | 0.02 | **Ind. pelagic longline** |  |
| Odontocetae | 108.90 | **Inshore gillnet curvina** |  | Lg. pelagic sharks | 997.29 |
| Extranjero | 65.11 | Drums & croakers | 4495.61 | Skates, rays & sharks | 79.63 |
| Barred pargo | 63.29 | Lg. Pelagics | 255.59 | Lg. pelagics | 57.02 |
| Sm. reef fish | 60.92 | Groupers & snappers | 98.48 | Sea birds | 0.00 |
| Lg. reef fish | 45.14 | Pinnipeds | 17.41 | **Demersal longlines** |  |
| Amarillo snapper | 39.54 | Barred pargo | 17.33 | Drums & croakers | 2520.12 |
| Lg. pelagics | 30.52 | Odontocetae | 16.53 | Groupers & snappers | 2066.49 |
| Gulf Coney | 20.61 | Gulf grouper | 14.16 | Gulf Coney | 1172.70 |
| Sea birds | 18.75 | Sea birds | 2.85 | Extranjero | 562.79 |
| Totoaba | 14.97 | Totoaba | 2.27 | Lg. pelagics | 170.25 |
| Hake | 10.57 | Mysticeti | 0.72 | Gulf grouper | 84.94 |
| Mysticeti | 4.74 | Reef associated turtles | 0.13 | Oceanic sea turtles | 45.69 |
| Oceanic sea turtles | 1.56 | Leopard grouper | 0.06 | Flatfish | 44.62 |
| Scorpionfish | 0.35 | Vaquita | 0.03 | Sm. reef fish | 32.21 |
| Vaquita | 0.22 | **Inshore gillnet other** |  | Reef associated turtles | 16.08 |
| Leopard grouper | 0.06 | Drums & croakers | 7492.69 | Hake | 10.57 |
| **Inshore demersal shark fleet** | | Herbivorous fish | 3336.82 | Lg. reef fish | 4.26 |
| Skates, rays & sharks | 3311.09 | Mojarra | 2425.28 | Barred pargo | 2.56 |
| Sm. migratory sharks | 1951.14 | Grunts | 912.96 |  |  |
| Lg. pelagic sharks | 276.85 | Sm. reef fish | 225.16 |  |  |
| Guitarfish | 33.25 | Pinnipeds | 51.36 |  |  |
| Pacific Angel shark | 4.02 | Odontocetae | 48.77 |  |  |

**Table S4 (continued)**

| **Fleet/ group caught** | **Catch (t)** | **Fleet/ group caught** | **Catch (t)** | **Fleet/ group caught** | **Catch (t)** |
| --- | --- | --- | --- | --- | --- |
| **Pelagic longlines** |  | **Fish traps (cont.)** |  | **Tuna purse seine** |  |
| Drums & croakers | 3101.68 | Lg. reef fish | 1615.13 | Lg. pelagics | 560.48 |
| Lg. pelagics | 397.25 | Sm. reef fish | 1505.42 | Lg. pelagic sharks | 12.40 |
| Groupers & snappers | 62.62 | Drums & croakers | 1411.16 | Sm. migratory sharks | 9.59 |
| Oceanic sea turtles | 0.17 | Herbivorous fish | 778.05 | Pacific Angel shark | 4.02 |
| Reef associated turtles | 0.06 | Skates, rays & sharks | 414.43 | Oceanic sea turtles | 0.03 |
| **Demersal handline** |  | Scorpionfish | 374.63 | **Sm. pelagic purse seine** |  |
| Groupers & snappers | 2800.39 | Extranjero | 115.26 | Sm. pelagics | 46902.73 |
| Extranjero | 1187.23 | **Compressor diving** |  | Hake | 190.20 |
| Carnivorous macrobenthos | 663.96 | Groupers & snappers | 1860.49 | Sm. reef fish | 32.21 |
| Lg. reef fish | 509.62 | Bivalves | 1650.85 | **Macroalgae** |  |
| Leopard grouper | 435.30 | Lg. reef fish | 1089.65 | Macroalgae | 306.63 |
| Sm. demersal fish | 254.64 | Snails | 658.03 | **Other gears** |  |
| Drums & croakers | 191.23 | Barred pargo | 121.34 | Drums & croakers | 225.00 |
| Barred pargo | 116.55 | Leopard grouper | 110.80 | Adult blue crab | 33.88 |
| Grunts | 61.85 | Drums & croakers | 106.65 | Skates, rays & sharks | 28.85 |
| Gulf grouper | 55.68 | Scallops & pen shells | 95.78 | Lg. pelagics | 22.79 |
| Herbivorous fish | 23.25 | Gulf grouper | 30.06 | Mojarra | 14.88 |
| Gulf Coney | 15.93 | Crabs & lobsters | 15.52 | Mackerel | 3.22 |
| Mojarra | 14.88 | **Octopus compressor diving** | | Penaeid shrimp | 2.12 |
| Amarillo snapper | 7.22 | Carnivorous macrobenthos | 135.38 | Leopard grouper | 0.56 |
| Sm. migratory sharks | 6.19 | **Penshell compressor diving** | |  |  |
| Lg. pelagics | 0.73 | Scallops & pen shells | 48.92 |  |  |
| Lg. pelagic sharks | 0.37 | **Sea cucumber compressor diving** | |  |  |
| Mackerel | 0.29 | Sea cucumbers | 276.42 |  |  |
| **Pelagic handline** |  | **Geoduck compressor diving** | |  |  |
| Sm. reef fish | 966.32 | Bivalves | 113.42 |  |  |
| Drums & croakers | 235.36 | **Jellyfish handnet** |  |  |  |
| Groupers & snappers | 84.86 | Jellyfish | 1655.90 |  |  |
| Lg. reef fish | 74.13 | **Recreational fishing** |  |  |  |
| Amarillo snapper | 7.22 | Groupers & snappers | 4471.53 |  |  |
| Herbivorous fish | 4.98 | Lg. reef fish | 2186.39 |  |  |
| Lg. pelagics | 0.77 | Drums & croakers | 739.38 |  |  |
| Mackerel | 0.68 | Extranjero | 367.31 |  |  |
| Lg. pelagic sharks | 0.37 | Sm. demersal fish | 280.70 |  |  |
| **Jaiba traps Kino** |  | Skates, rays & sharks | 48.50 |  |  |
| Adult blue crab | 1008.76 | Guitarfish | 45.98 |  |  |
| **Jaiba traps North of Desemboque** | | Flatfish | 44.62 |  |  |
| Adult blue crab | 1939.08 | Sm. migratory sharks | 29.73 |  |  |
| **Octopus traps** | | Barred pargo | 17.33 |  |  |
| Carnivorous macrobenthos | 8.54 | Mojarra | 14.88 |  |  |
| **Fish traps** |  | Leopard grouper | 14.12 |  |  |
| Groupers & snappers | 2228.60 | Lg. Pelagics | 7.63 |  |  |
| Sm. demersal fish | 1927.34 | Gulf grouper | 2.10 |  |  |

1. Pérez-Valencia S, Gorostieta-Monjaraz M, Castañeda-Fernández de Lara V, Loaiza-Villanueva R, Turk-Boyer P, et al. (2011) Manifestación de Impacto Ambiental para la pesca ribereña responsable en la Reserva de la Biosfera Alto Golfo de California y Delta del Río Colorado: Costa Este. Puerto Peñasco, Sonora: Centro Intercultural de Estudios de Desiertos y Océanos, A.C. 221 p.

2. Ainsworth C, Kaplan IC, Levin PS, Cudney-Bueno R, Fulton EA, et al. (2011) Atlantis model development for the Northern Gulf of California. NOAA Technical Memorandum NMFS-NWFSC-110. Department of Commerce. National Oceanic and Atmospheric Administration. National Marine Fisheries Service. Seattle, WA, USA. 293 p. Available:http://www.nwfsc.noaa.gov/assets/25/7784_08012011_125850_AtlantisModelTM110WebFinal.pdf. Accessed 2012 July 17.

3. Ainsworth CH, Morzaria-Luna H, Kaplan IC, Levin PS, Fulton EA (2012) Full compliance with harvest regulations yields ecological benefits: Northern Gulf of California case study. J Appl Ecol 49: 63–72. doi:10.1111/j.1365-2664.2011.02064.x.
