# Supplementary material for: Exploring Trade-Offs between Fisheries and Conservation of the Vaquita Porpoise (Phocoena sinus) Using an Atlantis Ecosystem Model
Source: PLoS One. 2012 Aug 15;7(8):e42917. doi: 10.1371/journal.pone.0042917 (PMC3419746; doi:10.1371/journal.pone.0042917)
Supplement: Table S5 — Catch for the shrimp light trawl fleet. Bycatch composition was based on INAPESCA & NMFS [1] and considering a reduction in shrimp catch of 10% [1] and an increase in the ratio of shrimp to bycatch (other than vaquita) of 11% [1], [2]. Vaquita bycatch was set to 0 for this fleet. (DOCX) [file pone.0042917.s011.docx]

| **Functional group** | **Catch (t)** |
| --- | --- |
| Penaeid shrimp | 771.78 |
| Flatfish | 86.30 |
| Drums and croakers | 83.73 |
| Sm. reef fish | 78.32 |
| Sm. demersal fish | 75.77 |
| Lg. Pelagics | 75.51 |
| Grunts | 72.84 |
| Groupers and snappers | 53.95 |
| Scorpionfish | 48.53 |
| Sm. Pelagics | 46.01 |
| Lg. reef fish | 27.01 |
| Mojarra | 24.29 |
| Skates, rays and sharks | 21.63 |
| Herbivorous fish | 16.19 |
| Guitarfish | 8.15 |
| Mackerel | 8.12 |
| Gulf Coney | 8.09 |
| Jellyfish | 5.60 |
| Carnivorous macrobenthos | 5.46 |
| Bivalves | 5.42 |
| Crabs and lobsters | 5.41 |
| Snails | 5.40 |
| Inf. epi. meiobenthos | 3.21 |
| Lanternfish and deep | 2.81 |
| Lg. pelagic sharks | 2.74 |
| Amarillo snapper | 2.70 |

1. INAPESCA, NMFS (2012) Fishing technology research collaboration: Efficiency and selectivity of two trawl nets design to fish blue shrimp (*Litopenaeus stylirostris*) in the artisanal shrimp fishery of the Upper Gulf of California. 9 p.

2. Pérez-Valencia S, Gorostieta-Monjaraz M, Castañeda-Fernández de Lara V, Loaiza-Villanueva R, Turk-Boyer P, et al. (2011) Manifestación de Impacto Ambiental para la pesca ribereña responsable en la Reserva de la Biosfera Alto Golfo de California y Delta del Río Colorado: Costa Este. Puerto Peñasco, Sonora: Centro Intercultural de Estudios de Desiertos y Océanos, A.C. 221 p.
