# Supplementary material for: Exploring Trade-Offs between Fisheries and Conservation of the Vaquita Porpoise (Phocoena sinus) Using an Atlantis Ecosystem Model
Source: PLoS One. 2012 Aug 15;7(8):e42917. doi: 10.1371/journal.pone.0042917 (PMC3419746; doi:10.1371/journal.pone.0042917)
Supplement: Table S6 — Sources for estimation of industrial fleets' cost rate of in the Northern Gulf of California. Average cost rate of 0.956 was used to calculate net present value. (DOCX) [file pone.0042917.s012.docx]

| **Cost rate** | **Fleet** | **Scale** | **Source** |
| --- | --- | --- | --- |
| 0.9898 | Shrimp trawl^1^ | National | [1] |
| 0.9515 | Shrimp trawl | National | [2] |
| 0.8945 | Shrimp trawl | Port (Guaymas, Sonora) | [3] |
| 0.99 | Small pelagic purse seine | State (Baja California) | [4] |

# ^1.^ Shrimp trawlers in the Northern Gulf also target finfish and giant squid during shrimp’s seasonal closure.

1. FIRA (2009) Situación actual y perspectivas del camarón en México. Morelia, Michoacán, México: Fideicomisos Instituidos en Relación con la Agricultura. 122 p. Available: http://www.fira.gob.mx/InfEspDtoXML/abrirArchivo.jsp?abreArc=3673. Accessed 2012 July 17.

2. FIRA (2002) Oportunidades para el desarrollo de la red de valor camarón. Morelia, Michoacán, México: Fideicomisos Instituidos en Relación con la Agricultura. p. Available: http://www.fira.gob.mx/InfEspDtoXML/abrirArchivo.jsp?abreArc=3438. Accessed 2012 July 17.

3. Ayala-Herrera GC (2001) Estructura e impacto de los subsidios en la pesquería industrial de camarón en el Golfo de California. El caso de la pesquería de camarón en el puerto de Guaymas [M.S.]. Hermosillo, Sonora: El Colegio de Sonora. 112 p.

4. CIDETE-UABC (2006) “Plan Maestro” del Sistema Producto de Pelágicos Menores de Baja California. Ensenada, B.C.: CONAPESCA-SAGARPA-UABC. 217 p. Available: http://www.conapesca.sagarpa.gob.mx/wb/cona/cona_programas_maestros. Accessed 2012 July 17.
