# Supplementary material for: Exploring Trade-Offs between Fisheries and Conservation of the Vaquita Porpoise (Phocoena sinus) Using an Atlantis Ecosystem Model
Source: PLoS One. 2012 Aug 15;7(8):e42917. doi: 10.1371/journal.pone.0042917 (PMC3419746; doi:10.1371/journal.pone.0042917)
Supplement: Table S7 — Price matrix for Atlantis functional groups. Values are dollars tonne-1 for 2010 or the most recent year for which data was available. For Penaeid shrimp, prices were set by fleet, weighted by the amount of blue, brown and Japanese shrimp caught. Information from National statistics for Sonora and Baja California, (Anuarios Estadísticos www.inegi.org.mx), state statistics for Sonora (www.oeidrus-sonora.gob.mx/), and port-level data for both states (unpublished data, A. Cinti, The University of Arizona, acinti@email.arizona.edu). Values were converted from Mexican pesos to dollars using the exchange rate from 2005–2010 (www.x-rates.com). (DOCX) [file pone.0042917.s013.docx]

| **Functional group** | **Values in dollars/ton** | **Year** |
| --- | --- | --- |
| Infaunal epifaunal meiobenthos | 17141.81 | 2008 |
| Scallops and pen shells | 16519.55 |  |
| Crabs and lobsters | 12586.32 |  |
| Penaeid shrimp |  |  |
| Shrimp driftnet Upper Gulf | 9265.25 |  |
| Shrimp driftnet Kino | 9265.25 |  |
| Other gears | 9265.25 |  |
| Industrial shrimp trawl Peñasco | 7292.72 |  |
| Industrial shrimp trawl Guaymas | 6891.71 |  |
| Herbivorous echinoderms | 4618.70 | 2008 |
| Carnivorous macrobenthos | 2828.46 |  |
| Amarillo snapper | 2740.69 |  |
| Groupers and snappers | 2740.69 |  |
| Hake | 2689.58 | 2008 |
| Gulf coney | 2073.14 |  |
| Barred pargo | 2005.95 |  |
| Extranjero | 1907.61 | 2008 |
| Sea cucumbers | 1807.03 | 2008 |
| Pacific Angel shark | 1605.21 | 2008 |
| Gulf grouper | 1601.49 | 2007 |
| Small reef fish | 1599.43 | 2008 |
| Flatfish | 1491.33 |  |
| Large reef fish | 1455.46 |  |
| Adult blue crab | 1413.36 |  |
| Bivalves | 1363.41 |  |
| Large pelagics | 1282.57 | 2008 |
| Small migratory sharks | 1218.45 |  |
| Large pelagic sharks | 1218.45 |  |
| Scorpionfish | 1162.47 | 2006 |
| Skates, rays and sharks | 960.93 |  |
| Snails | 895.78 |  |
| Small demersal fish | 796.01 |  |
| Guitarfish | 743.62 |  |
| Drums and croakers | 685.40 |  |
| Mojarra | 673.89 |  |
| Mackerel | 484.88 | 2009 |
| Herbivorous fish | 471.87 |  |
| Macroalgae | 449.77 | 2008 |
| Squid | 350.21 |  |
| Jellyfish | 78.66 |  |
| Small pelagics | 49.15 |  |

^1.^ Includes Abalone. ^2^. Grouped as sharks in statistics. ^3^. Grouped as cabrilla in statistics.
